# Novel Quantitative Autophagy Analysis by Organelle Flow Cytometry after Cell Sonication

**DOI:** 10.1371/journal.pone.0087707

**Published:** 2014-01-29

**Authors:** Michael Degtyarev, Mike Reichelt, Kui Lin

**Affiliations:** 1 Department of Translational Oncology, Genentech, South San Francisco, California, United States of America; 2 Department of Pathology, Genentech, South San Francisco, California, United States of America; Nottingham Trent University, United Kingdom

## Abstract

Autophagy is a dynamic process of bulk degradation of cellular proteins and organelles in lysosomes. Current methods of autophagy measurement include microscopy-based counting of autophagic vacuoles (AVs) in cells. We have developed a novel method to quantitatively analyze individual AVs using flow cytometry. This method, OFACS (organelle flow after cell sonication), takes advantage of efficient cell disruption with a brief sonication, generating cell homogenates with fluorescently labeled AVs that retain their integrity as confirmed with light and electron microscopy analysis. These AVs could be detected directly in the sonicated cell homogenates on a flow cytometer as a distinct population of expected organelle size on a cytometry plot. Treatment of cells with inhibitors of autophagic flux, such as chloroquine or lysosomal protease inhibitors, increased the number of particles in this population under autophagy inducing conditions, while inhibition of autophagy induction with 3-methyladenine or knockdown of ATG proteins prevented this accumulation. This assay can be easily performed in a high-throughput format and opens up previously unexplored avenues for autophagy analysis.

## Introduction

Macroautophagy (autophagy hereafter) is a well-conserved cellular catabolic process of self-degradation through the lysosomal machinery, and plays an important role in both normal physiology and diseases [1]. Autophagy is a complex and dynamic process, which is challenging to measure accurately [2], [3]. Commonly used methods to analyze autophagy include counting specific intracellular autophagic compartments that form during this process, using light microscopy or proper volumetric morphometry by electron microscopy.[2] For example, specific marker proteins attached to fluorescent tags such as mCherry-GFP-LC3B [4], or acidotropic dyes such as acridine orange (AO) or LysoTracker probes [2], [3], can be used to label autophagic or acidic compartments. Typically, image-based analysis is employed to analyze the fluorescent puncta observed under a microscope.

Microscopy analysis has certainly proven its value, but there are several disadvantages. Image acquisition and analysis are labor intensive and time consuming, prone to visual artifacts, and require large data storage space and expensive analysis softwares. In addition, it is often necessary to take multiple focus planes (z-sections) and fields, which require deconvolution to achieve unbiased measurement. As a result, microscopy analysis is relatively low throughput. Flow cytometry offers the advantage of analyzing a large number of cells on a cell-by-cell basis with more than 10 different fluorescent and light parameters available at the same time, but it lacks the capability to analyze intracellular structures, which is achievable with microscopy. To bridge this gap, we sought to develop an assay that could combine the advantages of both methods and apply it to measuring autophagy.

Whole cell flow cytometry has been previously described to monitor autophagy in a few publications,[4], [5], [6], [7], [8] which used whole-cell fluorescence intensity of AO or fluorescently tagged autophagy marker LC3B without counting individual AVs. In addition, FAOS (fluorescence-activated organelle sorting) has been described [9] as a method to sort labeled and gradient-purified organelles such as endosomes [10], or lysosomes, for which the term SOFA (single organelle flow analysis) has also been introduced [11]. The concept of “single organelle fluorescence analysis” was first used by Murphy's group to sort purified single organelles by flow cytometry [12]. Flow analyses of purified organelles, such as endosomes [13], mitochondria [14], phagosomes [15], and more recently autophagosomes and lysosomes [16], have been reported using various fluorescent probes. These reports relied on the established preparative methods for isolation and characterization of pure organelle fractions, including autophagosomes [11], [17], [18], [19], [20], which usually involve elaborate procedures that take several days, and are designed to isolate pure fractions from a single sample, usually starting from a large amount of material.

We have developed an assay aimed to achieve the following properties: easy to perform with a simple procedure, directly analyzing individual AVs both qualitatively and quantitatively, high throughput potential, using very limited sample amount, and applicable to measuring autophagy. In this report, we describe this novel quantitative method using flow cytometry to analyze AVs in crude cell homogenates directly after a brief sonication, which we termed OFACS (Organelle Flow After Cell Sonication).

## Results

### Sonication efficiently disrupted cells and released AVs that retained their integrity

Inhibition of the class I PI3K/Akt/mTOR pathway has been shown to activate autophagy [21], [22]. We employed two recently developed specific inhibitors of this pathway to generate cells with activated autophagy: the class I-selective PI3K (unless specified otherwise, PI3K refers to class I PI3K hereafter) inhibitor GDC-0941 [23] and the pan-Akt kinase inhibitor GDC-0068 [24]. Due to the dynamic nature of the autophagy flux the lifetime of the AVs can be very short and significant changes in AV numbers can be difficult to detect. To facilitate the detection of autophagic vacuoles, we also treated cells with the well-established inhibitors of autophagy flux, such as the lysosomotropic agent chloroquine (CQ) and lysosomal protease inhibitors, to prevent turnover and promote accumulation of AVs [21], [22].

Consistent with autophagy induction by inhibition of the PI3K/Akt/mTOR pathway, PC3 cells treated with the PI3K inhibitor GDC-0941 or the Akt inhibitor GDC-0068 alone showed a mild increase in acidic vesicles labeled by AO or LysoTracker Red observed under fluorescent microscopes (**Fig. S1A–C**). Co-treatment with the weak base CQ resulted in markedly increased accumulation of these vesicles, consistent with our previous reports [21], [22]. The majority of the acidic vesicles under GDC-0941 (or GDC-0068) and CQ co-treatment are likely AVs blocked by CQ at a stage that are acidic enough to be labeled with the acidotropic dyes, yet not acidic enough to complete the autophagic degradation. Western blot analysis of cell lysates after treatment by GDC-0941 and GDC-0068 showed increased degradation of p62 that was blocked by CQ, and the enhanced accumulation of LC3B-II in the presence of both the inhibitors and CQ, consistent with autophagic induction by the PI3K/Akt inhibitors that was blocked by CQ at the degradation step (**Fig. S1D**). The accumulation of LC3B-II and p62 could be detected as early as 3–6 hours and reached maximum between 24 and 48 hours, therefore most of our subsequent experiments were performed at 24 or 48 hours.

**Figure 1:**
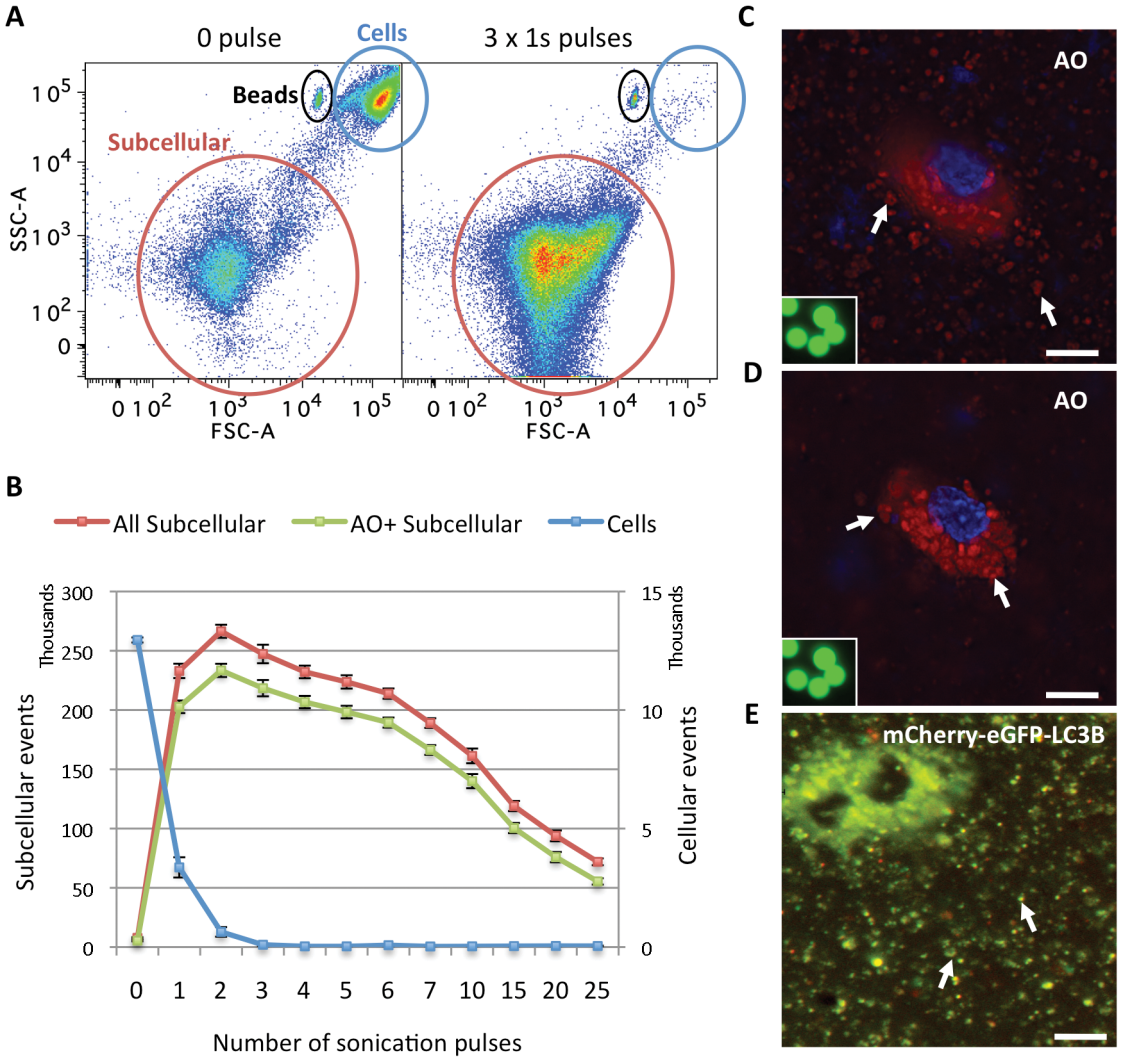
Sonication efficiently disrupted cells and released AVs that retained their integrity. (**A**) Forward scatter (FSC) vs. side scatter (SSC) plots of flow cytometric analysis of PC3 cell homogenates after a 2-day treatment with 1 µM GDC-0941 and 10 µM CQ, stained with AO and subjected to 0 (left) or 3 (right) 1s pulses of sonication. The position of the beads (7 µm) used as a standard for size threshold to distinguish cells from subcellular structures is circled in black. The distinct low FSC and SSC population circled in red, containing cell debris and organelles, is named the “subcellular” population, and used as such throughout this report. The high FCS and SSC population of intact cells is circled in blue. (**B**) Quantitative analysis of the number of total and AO^+^ subcellular events or cells in the corresponding populations defined in (**A**) as a function of the number of 1s sonication pulses. Error bars represent standard errors of mean (SEM), n = 7. Representative data from 2 independent experiments are shown. (**C,D**) Microscopy images of PC3 cells stained with AO and Hoechst 33342 and sonicated with 3 x 1s pulses showing the bottom focus plane with released vacuoles in focus (**C**) and the mid-cell range focus plane with an unbroken cell in focus (**D**). RGB images obtained in ex FITC/em Cy5 and ex DAPI/em DAPI channels are merged using Adobe Photoshop. Inset: 7 µm beads in the green (em FITC/ex FITC) channel under microscope at the same magnification. Examples of AVs are indicated with white arrows. Scale bars: 20 µm. (**E**) Microscopy image of PC3 cells stably expressing mCherry-eGFP-LC3B co-treated with 1 µM GDC-0941 and 10 µM CQ for 2 days and sonicated with 3 x 1s pulses. Two unbroken cells in the upper left corner filled with AVs are out of focus. Mostly “yellow” (“red” and “green” dual positive) AVs on the bottom of the plate are in focus. RGB images from red (mCherry) and green (eGFP) channels are merged using Adobe Photoshop. Examples of AVs are indicated with white arrows. Scale bar: 20 µm.

To open up the cells and release the vacuoles, PC3 cells treated with GDC-0941 and CQ were stained with AO and then subjected to sonication for an increasing number of 1-second (1s) pulses. Cell homogenates were then analyzed using a flow cytometer. As seen on a forward scatter (FSC) vs side scatter (SSC) plot (**Fig. 1A** **& Fig. S1E & F**), three 1s pulses of sonication efficiently disintegrated the cells and produced a distinct population with an estimated size range of organelles about 100–1000 times smaller than the population of intact cells. As shown in **Fig. 1B**, the number of organelles reached maximum and is stable within a range of 1–5 pulses. Meanwhile, there was a corresponding drop in the number of intact cells as the number of organelles increased. AO is a weak base that moves freely across biological membranes when uncharged. Its protonated form accumulates in acidic compartments, where it forms aggregates that fluoresce bright red, whereas the cytoplasm and the nucleus showed dominant green fluorescence. Microscopy analysis of cells that were stained with AO followed by 3 x 1s pulses of sonication confirmed the release of vacuoles that maintained their integrity as suggested by their retention of the red fluorescent AO staining after sonication, similar to those inside an unbroken cell (**Fig. 1C vs D**). Microscopy analysis of cells lysed after labeling with LysoTracker Green and sonication (**Fig. S2A**) also confirmed the release of labeled intact vacuoles after sonication.

**Figure 2:**
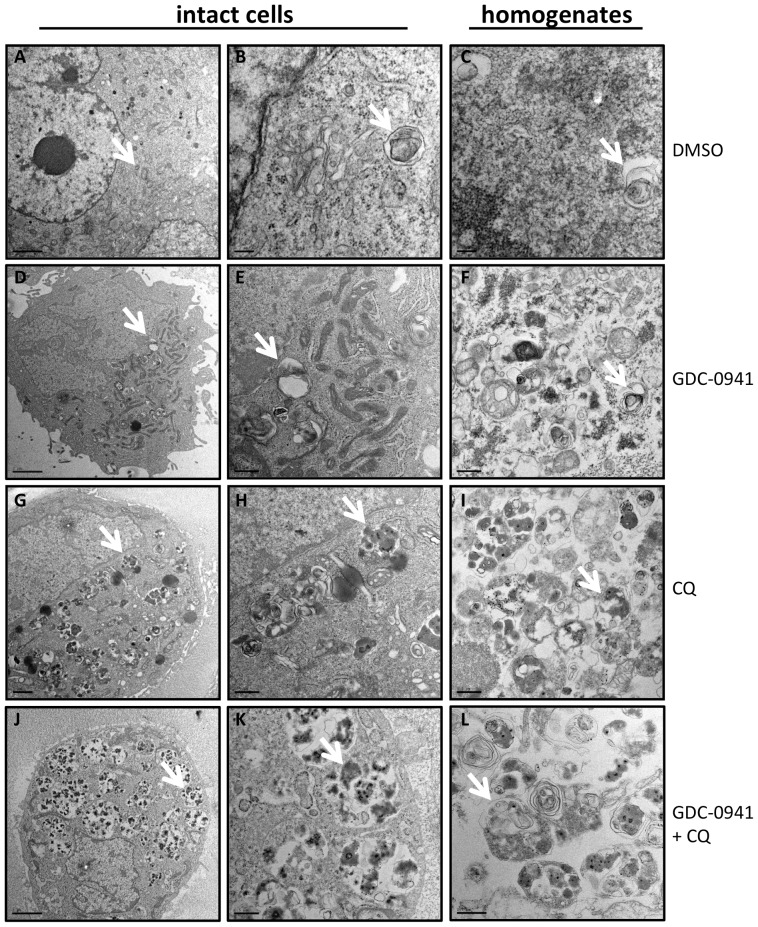
TEM analysis of subcellular structures in both intact and 3 x 1s sonicated homogenates of PC3 cells. (**A–C**) PC3 cells treated with DMSO showed only occasional autophagosome and autolysosome structures in both intact cells and homogenates. (**D–F**) PC3 cells treated with 2 µM GDC-0941 for 24 hours showed a slightly increased number of autophagosome and autolysosome structures in both intact cells and homogenates compared to (**A**). (**G–I**) PC3 cells treated with 10 µM CQ contained increased number of AVs mostly of autolysosomal nature in both intact cells and homogenates. (**J–L**) PC3 cells treated with both GDC-0941 and CQ are packed with AVs mostly of autolysosomal nature in both intact cells and homogenates. Arrows indicate representative AV structures in each population; scales bars: 2 µm (A,D,G,J), 0.2 µm (B,C) & 0.5 µm (E,F,H,I,K,L).

Since AO can stain acidic vesicles other than AVs, we also analyzed PC3 cells stably expressing a pH-sensitive autophagy reporter mCherry-eGFP-LC3B [25]. Treatment of these cells with GDC-0941 or GDC-0068 alone induced an increased number of red puncta, which represent the acidic autophagolysosomes (autophagosome-lysosome fusion) and amphisomes (autophagosome-endosome fusion) due to the acid-labile feature of GFP but not mCherry, indicating rapid dynamics of the autophagic flux induced by these treatments (**Fig. S2B,C**). In the presence of CQ, these treatments induced a strong accumulation of vacuoles that fluoresced both green and red and appeared “yellow” in the merged images taken under a fluorescence microscope, representing AVs of lower acidity due to inhibition of the acidification and fusion of autophagosomes with lysosomes or endosomes by CQ [16]. PC3 expressing cells mCherry-eGFP-LC3B were treated with GDC-0941 and CQ and subjected to sonication as described above. As shown in **Fig. 1E**, sonication released AVs that retained their fluorescence properties in the cell homogenate. Finally, transmission electron microscopy (TEM) analysis of intact and sonicated cell homogenates revealed that the homogenates consists of autophagic vacuoles that appear very similar to the structures in intact cells (**Fig. 2**). 3 x 1s sonication was therefore chosen as a standard method for all subsequent studies.

### Pharmacologically induced AVs can be individually detected by OFACS from sonicated cells stained with AO or other acidotropic dyes

**Figure 3:**
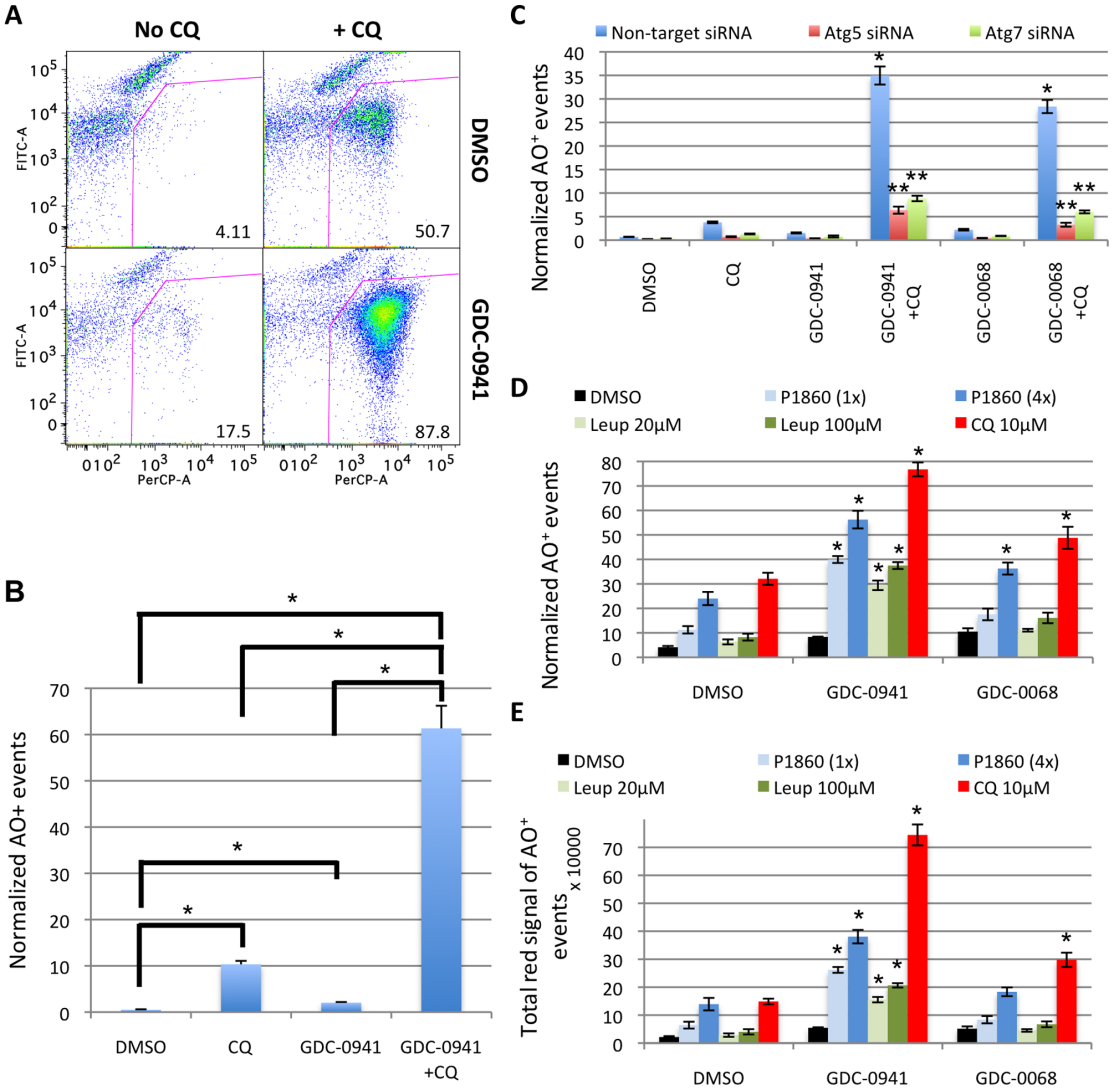
Pharmacologically induced acidic vacuoles can be individually detected by OFACS from sonicated cells stained with AO. **(A)** PC3 cells treated with 1 µM GDC-0941 and 10 µM CQ for 2 days analyzed by OFACS after staining with AO. FITC (green) vs. PerCP (red) channels of the organelle population are plotted. Percentage of the gated AO^+^ population is shown for each plot. **(B)** Quantification of the number of AO^+^ events as gated in **(A)** normalized to the number of cells used for sonication with each treatment. *, P<0.05. **(C)** Quantitative analysis by OFACS showing the normalized number of AO^+^ events under indicated treatments. PC3 cells transfected with Atg5 or Atg7 siRNA for 2 days were treated with 1 µM GDC-0941 or 5 µM GDC-0068 +/− 10 µM CQ for an additional day. *, P<0.05 vs. DMSO, CQ or GDC-0941/GDC-0068 alone with non-target siRNA; **, P<0.05 vs. non-target siRNA in the same treatment group. **(D,E)** Quantification of AO^+^ events obtained by OFACS analysis of PC3 cells treated with GDC-0941 or GDC-0068 +/− CQ or protease inhibitors for 2 days, showing the normalized number of AO^+^ events **(D)** and the total “red” signal intensity of AO^+^ events **(E)**, derived by multiplying the number of AO^+^ organelles by the mean value of the “red” AO signal. *, P<0.05 both vs. DMSO group treated with the same lysosomal inhibitor and vs. GDC-0941 or GDC-0068 alone. Error bars represent SEM (n = 3). P values are determined by Student's t-test. Representative data from one of three independent experiments are shown. All quantifications are normalized to the number of cells used for the sonication under each condition.

Flow cytometric analysis of the cell homogenates from PC3 cells treated with GDC-0941 and CQ for 2 days revealed a dramatic increase in the AO-stained population (**Fig. 3A,B**), consistent with microscopy observations (**Fig. S1A**). When plotted by the signals detected in the FITC (green) vs PerCP (red) channels, the subcellular population, as defined in **Fig. 1A**, showed an increased percentage of particles with high “red” and low “green” signals in the cells treated with both GDC-0941 and CQ compared to “no drug” control, GDC-0941 or CQ alone groups (**Fig. 3A,B**), consistent with an increase in the accumulation of AO^+^ AVs induced by this treatment. GDC-0941 treatment alone also caused a small increase in this population, consistent with an increased flux to acidic compartments resulting from increased autophagy. Similar results were obtained with the Akt inhibitor GDC-0068 and CQ (**Fig. 3C**). This accumulation of AO^+^ subcellular events is greatly reduced by the knockdown of Atg5 and Atg7 genes, which are crucial for the formation of AVs (**Fig. 3C** **& Fig. S3**).[2] Detection of the AO^+^ event accumulation by OFACS could also be observed with other late-stage autophagy inhibitors such as the lysosomal protease inhibitor leupeptin or a protease inhibitor cocktail P1860 (**Fig. 3D,E**). This effect was dependent on the concentration of the inhibitors and observed for both PI3K and Akt inhibitors, with 10 µM CQ showing the strongest effect among the agents tested. This may reflect the general inhibition of lysosomal proteases by CQ due to its acidotropic effect, compared to the more selective activities of the other protease inhibitors.

Remarkably, four related readout outputs: 1) normalized total number of “subcellular” events (all events were normalized to cell number used in the sonication); 2) normalized number of AO^+^ or LysoTracker Red^+^ subcellular events; 3) total “red” channel intensity (red signal) of AO^+^ subcellular events; and 4) percentage of AO^+^ subcellular events of the total “subcellular” population showed very similar results qualitatively (**Fig. S4A–E**). The “total signal intensity” parameter provides a three-dimensional volume measurement of the fluorescence signal from all labeled organelles, which would normally require z-series and de-convolution procedures to approximate by conventional microscopy. The “percentage of AO^+^ events” of the total “subcellular” population parameter does not require cell number normalization, and therefore could be more useful in a high-throughput setting. Interestingly, these data suggest that the total normalized number of the “subcellular” events measured in this assay can be used as an independent parameter to characterize the degree of autophagy in the absence of a specific marker under conditions known to induce accumulation of AVs, when the majority of organelles accumulated under these conditions are AVs, which should be verified using classical assays such as EM or light microscopy.

Similar results were obtained in a different cell line, HEK293, where we also compared the use of AO to LysoTracker Red in labeling acidic vesicles (**Fig. S4F–H**). The normalized numbers of dye-positive vacuoles were very close for the two dyes.

This assay was easily adaptable to 96 or 384-well format, allowing high throughput measurements such as time-course treatments, concentration curves, determination of combination effects of drug treatment that can be calculated using the bliss analysis [26] (**Fig. S4I–K & Fig. S5A–D**).

### Pharmacologically induced AVs can be individually detected by OFACS using cells expressing fluorescently tagged LC3B protein

**Figure 4:**
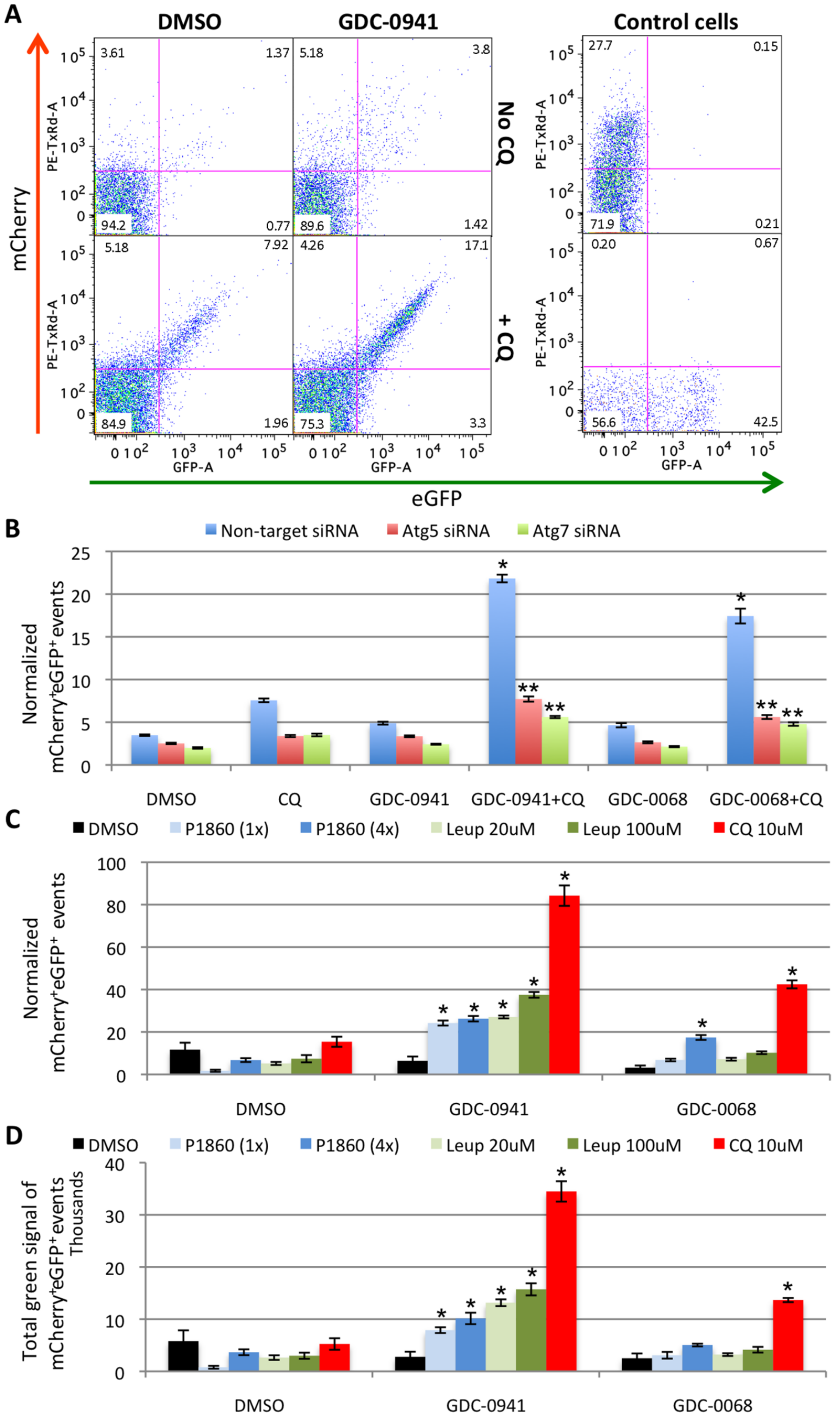
Pharmacologically induced AVs can be individually detected by OFACS from sonicated PC3 cells expressing mCherry-eGFP-LC3B. **(A)** mCherry (ex PE/em Texas Red, “red” channel) vs. eGFP (FITC “green” channel) plots showing accumulation of the mCherry^+^eGFP^+^ organelle population in PC3 cells co-treated for 2 days with 1 µM GDC-0941 and 10 µM CQ vs. each drug alone or no drug. Control PC3 cells expressing free mCherry (top) or eGFP-LC3B (bottom) treated with GDC-0941 and CQ analyzed the same way are shown on the right as single channel controls. Numbers in upper right quadrants represent the percentage of mCherry^+^eGFP^+^ events of the total subcellular population. **(B) **Normalized number of mCherry^+^eGFP^+^ events in cells treated with the indicated agents and the effects of Atg5 or Atg7 siRNA on this population. *, P<0.05 vs. DMSO, CQ or GDC-0941/GDC-0068 alone with non-target siRNA; **, P<0.05 vs. non-target siRNA in the same treatment group. **(C,D)** Normalized number of mCherry^+^eGFP^+^ AVs per cell **(C)** or total GFP intensity of the mCherry^+^GFP^+^ AVs **(D)** in PC3 cells co-treated with GDC-0941/GDC-0068 and protease inhibitors or CQ as indicated. *, P<0.05 both vs. DMSO group treated with the same lysosomal inhibitor and vs. GDC-0941 or GDC-0068 alone. PC3 cells stably expressing the mCherry-eGFP-LC3B marker were treated with 1 µM of GDC-0941 **(A–D)**, or 5 µM of GDC-0068 **(B-D)** +/− 10 µM CQ or the indicated concentrations of protease inhibitors for 1 day, sonicated and analyzed by OFACS. Error bars represent SEM (n = 4). Representative data from one of three independent experiments are shown.

To date, the LC3B protein represents the best-characterized specific marker for autophagosomes [2], [3]. Here, we show that the pH-sensitive fluorescently tagged mCherry-eGFP-LC3B protein can be used as a marker for AV detection by OFACS (**Fig. 4**). Consistent with microscopy images of cells treated with GDC-0941 or GDC-0068 +/− CQ shown in **Fig. S2B**, combined treatment with GDC-0941 and CQ increased the percentage of the organelles positive for both mCherry and eGFP fluorescence (**Fig. 4A**) and the normalized number of double positive events (**Fig. 4B**). Similar effect was observed with GDC-0068. This could be effectively prevented by knockdown of Atg5 or Atg7 with siRNA, similar to that observed with AO staining (**Fig. 4B**). In addition, CQ-mediated double positive event accumulation could be phenocopied with other lysosomal protease inhibitors (**Fig. 4C,D**). Similarly, when the singly fluorescence-tagged eGFP-LC3B was used as a marker, GDC-0941 and CQ co-treatment also induced strong accumulation of eGFP^+^ AVs that can be readily detected by OFACS (**Fig. S6A–C** & **Fig. 4A** bottom control cells). We also used singly RFP-labeled LC3B as a marker, and compared that to a lipidation-defective mutant of LC3B, LC3B-G120A [27]. As expected, LC3B-F120A-RFP failed to form AV puncta when compared to wild-type LC3B-RFP in PC3 cells treated with GDC-0941 and CQ, and did not form the RFP^+^ subcellular population that could be detected with the wild-type LC3B-RFP (**Fig. S7A–D**). These data demonstrate the specificity of the RFP^+^ population defined by the OFACS assay as autophagic vacuoles. OFACS can also be used to measure autophagy activity in response to classic stimuli such as starvation in HBSS and treatment with the mTOR inhibitor rapamycin (**Fig. S8**). As has been shown by others, rapamycin induced accumulation of AVs at a very low concentration but plateaued early at a lower level of AV induction than the PI3K inhibitor GDC-0941, consistent with its incomplete inhibition of mTOR activity [28].

When the OFACS experiments were run in parallel with microscopy image analysis, high degree of positive correlation was observed between the microscopy and the OFACS quantifications (Table S1 & Fig. S6E). Notably, the OFACS method could accurately quantify AVs even when the cells were fully packed with these vacuoles, such as when cells were treated with GDC-0941 or GDC-0068 and CQ for 12–24 hours, while the microscopy counting software was unable to distinguish between individual vacuoles within the overlapping dots or clusters of dots under these conditions.

We also explored the application of OFACS protocol to multispectral imaging flow cytometry (MIFC) using an ImageStream imaging flow cytometer (**Fig. S9**). Whole cell MIFC analysis revealed the presence of puncta with green (eGFP) and red (mCherry) fluorescence in PC3 cells treated with GDC-0941 +/− CQ, but lacked the detailed information and the ability to accurately count the number of fluorescent spots inside cells (**Fig. S9A**). Sonication enabled the analysis of individual AVs with multiple parameters and concomitant visualization of the particles in both fluorescent channels and brightfield (**Fig. S9B,C**). Comparison of the mCherry^+^eGFP^+^ AV numbers obtained by MIFC vs conventional flow cytometry revealed highly comparable results by the two methods (**Fig. S9D**). In addition, MIFC assay indicated that about 99% of the particles in the sonicated cell homogenate are single organelles, with under 0.7% of organelle doublets. (**Fig. S9C**).

### Inhibition of autophagy by 3-methyladenine at an early stage and by Bafilomycin A1 at a later stage can be distinguished by OFACS analysis of individual AVs

**Figure 5:**
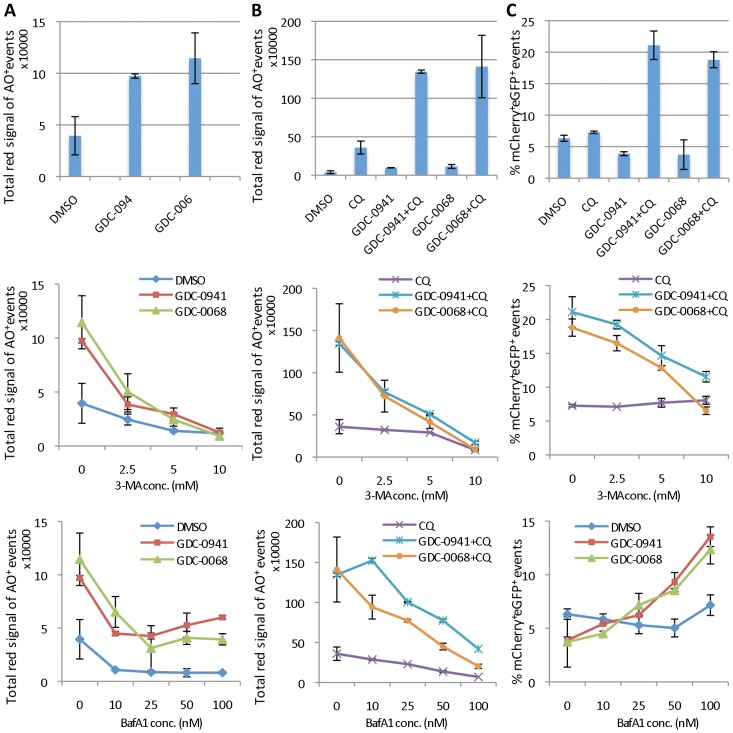
Inhibition of autophagy by 3-MA at an early stage and by BafA1 at a later stage can be distinguished by individual AV analysis with OFACS. Untransfected PC3 cells **(A,B)** or PC3 cells expressing mCherry-eGFP-LC3B **(C)** treated with 1 µM GDC-0941 or 5 µM GDC-0068 +/− 10 µM CQ for 24 hours with the addition of: nothing (upper panels), 0–10 mM 3-MA (middle panels) or 0–100 nM Bafilomycin A1 (BafA1; bottom panels). AVs were analyzed by OFACS after staining with AO **(A,B)** or unstained **(C)**. Y axes represent the total “red” channel intensity of AO^+^ organelles normalized to cell number **(A,B)** or the percentage of mCherry^+^eGFP^+^ events of the total subcellular events **(C)**. Note the different scales of the y-axes in** (A)** vs.** (B).** Error bars represent standard deviations (SD), n = 3.

3-methyladenine (3-MA) is an inhibitor of class III PI3K that is necessary for early AV formation. It is widely used to inhibit autophagy at an early stage.[29] Bafilomycin A1 (BafA1), on the other hand, is an inhibitor of the vacuolar-type H^+^-ATPase and inhibits autophagy at a later stage similar to CQ, by inhibiting the acidification of AVs resulting from fusion of autophagosomes with endosomes and/or lysosomes [30]. The accumulation of AO^+^ organelles or mCherry^+^eGFP^+^ LC3B AVs, induced either by GDC-0941/GDC-0068 alone or in combination with CQ (**Fig. 5A–C** upper panels), was inhibited in a concentration-dependent manner by 3-MA as detected by the OFACS analysis (**Fig. 5A–C** middle panels). BafA1 reduced the accumulation of AO^+^ events as expected, since it prevents the acidification of the lysosomal compartment likely more strongly than the concentrations of CQ used here, so that AO can no longer label these vesicles, and thus reducing AO staining in treatments with and without CQ (**Fig. 5A,B** bottom panels). On the other hand, BafA1 increased accumulation of mCherry^+^eGFP^+^ AVs in mCherry-eGFP-LC3B expressing cells treated with GDC-0941 and GDC-0068, as expected from its inhibition of autophagosome-endosome/lysosome fusion and autophagic degradation, similar to the effect of CQ (**Fig. 5C** bottom panel). The fact that OFACS is able to distinguish between the early and late autophagy inhibitors with different mechanisms of action further demonstrates the power of the technique.

### Non-specific autophagic bulk protein accumulation in AVs can be detected by the OFACS assay

To prove the concept of using OFACS assay to detect autophagic bulk protein accumulation in individual AVs we overexpressed the mCherry protein alone in PC3 cells stably expressing eGFP-LC3B. Two days after transfection cells were treated with GDC-0941 or GDC-0068 +/− CQ or protease inhibitors for 24 hours and analyzed by the OFACS assay. As expected, strong accumulation of eGFP^+^ AVs was observed when GDC-0941 or GDC-0068 was combined with CQ and to a lesser extent with protease inhibitors, while 3-MA completely abolished AV accumulation (**Fig. S6C**). Interestingly, about 50% of the GFP^+^ vesicles were also mCherry^+^ (**Fig. S6D**). Thus, mCherry as a non-specific exogenously overexpressed protein could be captured by the eGFP-LC3B^+^ AVs and detected by the OFACS assay. In fact, the accumulation of mCherry^+^ organelles could also be detected when transfected into cells without the expression of eGFP-LC3B (**Fig. 4A** top control cells).

### OFACS detected colocalization of fluorescently tagged LC3B, p62, and AO with fluorescently labeled chloroquine in AVs

**Figure 6:**
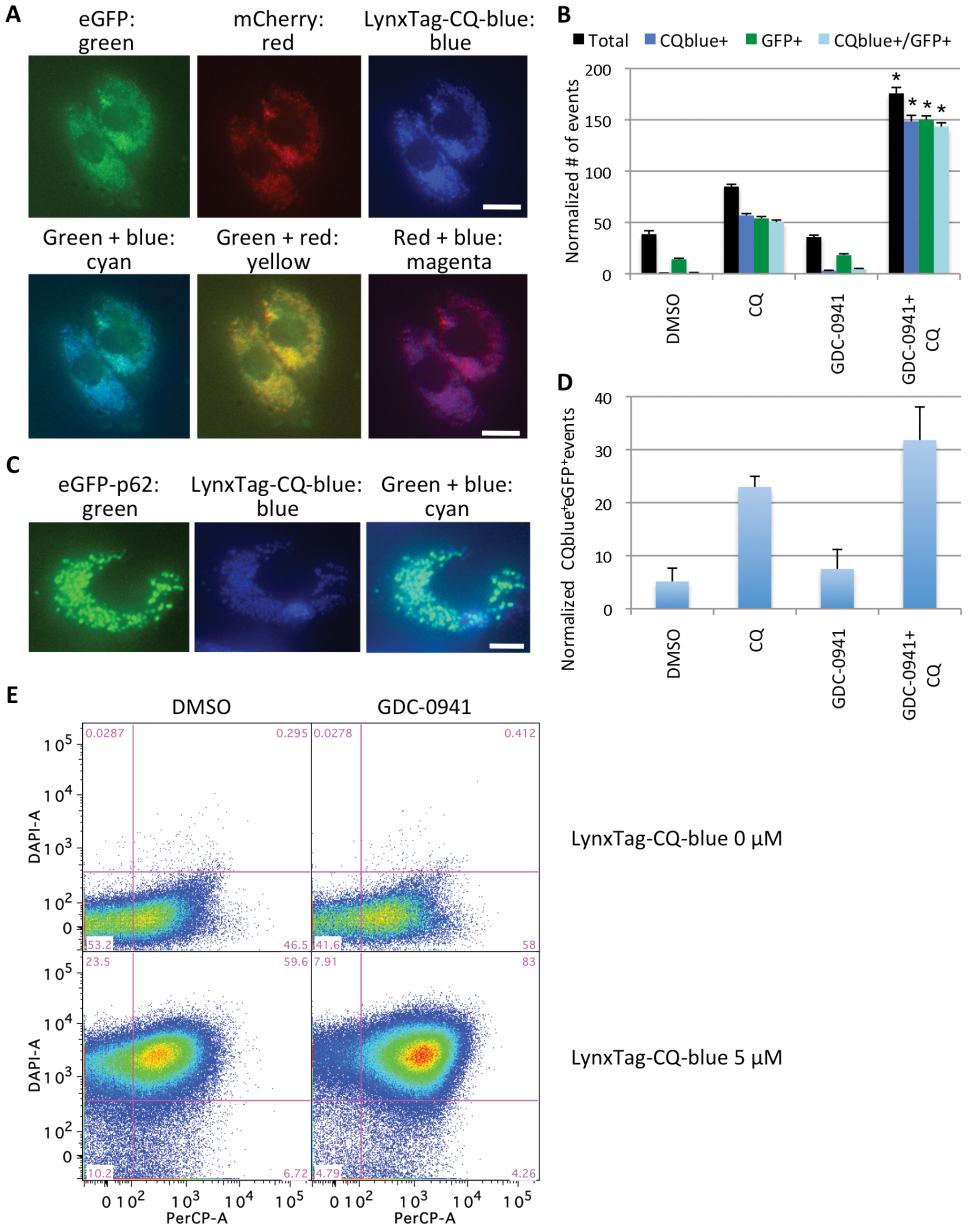
mCherry-eGFP-LC3B, eGFP-p62, and Acridine Orange are co-localized with fluorescently-labeled chloroquine in AVs detected by OFACS. **(A)** Representative microscopy images of PC3 cells stably expressing mCherry-eGFP-LC3B and treated with 1 µM GDC-0941 +/− 9 µM CQ and 1 µM LynxTag-CQ-blue for 24 hours. Images are shown in each fluorescence channel separately (top panels) or merged (bottom panels). Scale bar: 20 µm. **(B)** OFACS quantification of the experiment in **(A)** showing the normalized numbers of events with the indicated labels: total normalized number of “subcellular” events (black bars), CQblue^+^ (blue bars), eGFP^+^ (green bars) and dual positive (CQblue^+^eGFP^+^) (cyan bars). *, P<0.05 vs. DMSO, CQ or GDC-0941 only groups. **(C)** Representative microscopy images of PC3 cells transiently transfected with eGFP-p62 for 48 hours and treated with GDC-0941 (1 µM) +/− 9 µM CQ and 1 µM LynxTag-CQ-blue for 24 hours. Images are shown in green or blue channels separately or merged. Scale bar: 10 µm. **(D)** OFACS quantification of the experiment in **(C) **showing the normalized number of CQblue^+^eGFP^+^ events under each treatment. **(E)** “Red” (PerCP) vs. “blue” (DAPI) channel plots of PC3 cells treated with 1 µM GDC-0941 +/− 5uM LynxTag-CQ-blue for 24 hours, stained with AO, sonicated and analyzed by OFACS. Error bars represent SEM (n = 4). Representative data from 2 independent experiments are shown.

To further confirm that the subcellular events detected with AO or fluorescently tagged AV markers by the OFACS assay could indeed be used to quantity autophagy induced with the PI3K/Akt inhibitors and accumulated with CQ treatment, we performed co-localization studies with fluorescently tagged CQ. First, we observed that similar to unlabeled CQ, LynxTag-CQ-blue promoted GDC-0941-induced accumulation of AVs but these AVs are now fluorescent in the blue (DAPI) channel due to the accumulation of LynxTag-CQ-blue in them (**Fig. S10A**). Second, we confirmed that transiently transfected eGFP-p62 behaved as an AV marker, accumulating in GDC-0941 and CQ co-treated cells, consistent with the role of p62 in delivering ubiquitinated proteins into autophagosomes via binding to LC3 family members (**Fig. S10B**) [31]. Finally, when PC3 cells expressing mCherry-eGFP-LC3B (**Fig. 6A,B**) or PC3 cells transfected with eGFP-p62 (**Fig. 6C,D**) were treated with GDC-0941 +/− CQ with spiked-in LynxTag-CQ-blue, the fluorescently tagged autophagy marker proteins co-localized with fluorescent CQ, revealed by both microscopy images of intact cells and the OFACS assay. A similar pattern of co-localization was observed with PC3 cells treated with GDC-0941 +/− LynxTag-CQ-blue and stained with AO (**Fig. 6E** **; Fig. S11**).

We also evaluated another lysosomotropic agent quinacrine (QC) and compared it to CQ both under the microscope and in the OFACS assay (**Fig. S10C,D**). Of note, we found that quinacrine was very strongly autofluorescent in green (FITC) channel and could be used as an acidic vesicle dye. In addition, quinacrine was about 5∼10-fold more potent than CQ in inducing AV accumulation when combined with GDC-0941 (**Fig. S10E**).

Using RFP-tagged organelle-specific membrane markers, we evaluated the contributions of membranes from other organelles to the “subcellular” population detected by OFACS, including plasma membrane, nuclear, endoplasmic reticulum, mitochondria, and the Golgi apparatus. To exclude organelles that have been enclosed by an AV, we stained the cells with QC and quantitated RFP^+^QC^-^ events. The results suggest that under non-autophagy inducing conditions, these labeled organelles or their fragments each contributes 10–40% of the total subcellular events detected, while in cells treated with both GDC-0941 and CQ, each of these contributes less than 5% of the total subcellular events, consistent with the degradation-arrested AVs make up the majority of the subcellular events under this condition (**Fig. S12**)

Finally, we explored flow cytometric sorting of specifically labeled AVs from the sonicated cell homogenates. After a single sort, AVs induced by GDC-0941 and CQ/CQ-blue co-treatment that are labeled with mCherry-eGFP-LC3B and CQ-blue could be significantly enriched as indicated by the greatly increased percentage of events in the non-specific “organelle” population as well as the specific dual CQblue^+^mCherry^+^ or triple CQblue^+^mCherry^+^GFP^+^ populations (**Fig. S13**).

## Discussion

The OFACS analysis of sonicated crude cell homogenates by flow cytometry, described here, represents a conceptually different approach from the previously described methods to analyze autophagy. The assay is simple yet highly quantitative. From serial dilution experiments, we have found that AO^+^ organelles can be detected with accuracy from 1–5 cells, depending on the number of AVs in the cells. To our knowledge, this is the first report showing flow cytometry-based quantitative and qualitative analysis of individual AVs from a small amount (as little as 10 µL) of unpurified, sonicated cell homogenates, using specific organelle markers and dyes. This method helps to overcome some limitations of microscopy and offers advantages that the flow cytometry technology provides, is amenable to high-throughput, and opens new opportunities for autophagy research.

EM and light microscopy analysis of sonicated cells showed that the released AVs retained label and size comparable to those inside intact cells. The bell shape of the organelle formation curve upon sonication also suggests that they maintain their integrity and number during the first few pulses. Only after further sonication (**Fig. 1B**) or 0.1% Triton X-100 treatment did we observe disintegration of AVs' membrane and release of the dye (data not shown), suggesting that these accumulated AVs are fairly resilient to mild mechanical disruption. Although we could not find existing data on the stability of lysosomes and AVs under sonication, we speculate that the dense content and double- or multi-membrane nature of autophagosomes and amphisomes [32] might make them more stable compared to other single-membrane organelles, such as lysosomes. For example, multilamellar liposomes form more readily with sonication and thus might be more thermodynamically stable [33]. One of the advantages of sonication could be that it likely untangles the AVs from the intracellular microtubule and cytoskeletal networks, allowing their individual analysis by flow cytometry.

In summary, using a novel flow cytometric analysis of organelles released from cells after a brief sonication, we have confirmed and carefully validated a specific and distinct subcellular population. This population was detected upon autophagy induction in different cell types, could be labeled with specific autophagosome markers including eGFP-LC3B, mCherry-eGFP-LC3B and eGFP-p62, as well as non-specifically sequestered, overexpressed mCherry protein, and stained with Acridine Orange and other acidotropic dyes or fluorescent CQ. The results were reproducible with different autophagy inducers (including different PI3K/Akt/mTOR pathway inhibitors and starvation), confirmed with various autophagy inhibitors (Atg knockdowns, 3-MA, BafA1, CQ, lysosomal protease inhibitors, quinacrine), consistent with microscopy analysis, and applicable to both conventional flow cytometry and multispectral imaging flow cytometry. Of course, as with any other methods for autophagy characterization, additional confirmation with parallel methods is always needed to confirm the nature of the autophagic response.

One can envision using the OFACS assay with any other newly discovered fluorescently tagged AV markers. Characterization and knowledge of additional specific markers on the outer surface of AVs and the availability of antibodies against them should allow us to further test the utility of this method for AV detection and characterization in the future. Further studies are warranted to apply this assay to the in vivo setting, either employing fluorescent dyes or specific antibodies against AV markers. For example, AO could be used to stain post-sonication homogenates of tissues and then analyzed by OFACS. Additional work is also needed to optimize this assay to sort distinct fractions of organelles based on different markers. This assay offers the advantage of flow cytometry technology, and could help resolve current controversies in autophagy research, such as distinguishing between inner and outer surface of autophagosomes, searching for additional markers of AVs, or finding the origin of the autophagic membrane.

## Materials and Methods

### Materials

Protease inhibitor cocktail P1860 (Sigma, 1x equals to 500 fold dilution of the stock solution) and P-8340 (Sigma, 1x equals to 2000 fold dilution of the stock solution), Leupeptin (Sigma), 3-MA (Sigma), Bafilomycin A1 (Sigma), Acridine Orange (AO) (Sigma), LysoTracker probes (Invitrogen), chloroquine diphosphate (CQ) (Fluka), CountBright™ fluorescent beads (Invitrogen, C36950), PI3K inhibitor GDC-0941 and Akt inhibitor GDC-0068 (Genentech), human LC3B conjugated to eGFP [21] and mCherry-eGFP expression construct (Genentech), eGFP tagged human p62 construct (Genentech), Quinacrine (Sigma), LynxTag-CQ green and blue (BioLynx Technologies, Singapore), Hoechst 33342 (Invitrogen), Lipofectamine RNAiMAX and Lipofectamine 2000 (Invitrogen), Atg5 antibody (Abgent, catalog# AP1812b), Atg7 antibody (Santa Cruz Biotech, catalog# sc-33211), GAPDH antibody (Advanced ImmunoChemical, catalog# RGM2).

### Cell staining with fluorescent dyes

Cells were incubated for 30–60 minutes with AO (0.1 µg/ml), LysoTracker Red or Green (0.1 µg/ml), Hoechst 33342 (1 µg/ml), quinacrine (1 µM) in growth media in a 37°C 5% CO_2_ tissue culture incubator or in PBS buffer containing 0.1% BSA on ice.

### Transmission Electron Microscopy

All samples (cells and homogenates) were fixed in modified Karnovsky's fixative (2% paraformaldehyde and 2.5% glutaraldehyde in 0.1 M sodium cacodylate buffer, pH7.2). The pellets of the homogenate samples were stabilized by mixing with 10% gelatin. All samples were post-fixed in 1% aqueous osmium tetroxide for 2 hours and then dehydrated through a series of ethanol (50%, 70%, 90%, 95%, 100%) followed by propylene oxide (each step was for 15 min) and embedded in Eponate 12 (Ted Pella, Redding, CA). Ultrathin sections (70 nm) were cut with an Ultracut microtome (Leica), stained with 3.5% aqueous uranyl acetate and 0.2% lead citrate and examined in a JEOL JEM-1400 transmission electron microscope (TEM) at 120 kV. Digital images were captured with a GATAN Ultrascan 1000 CCD camera.

### OFACS protocol for direct detection of individual AVs

Flow cytometry data were acquired with BD LSR-II or BD LSRFortessa (BD Biosciences) using the HTS auto-sampler device, and sorting was performed on a BD FACS Aria2 using excitation lines at 488 nm and 561 nm and detecting fluorescence at 530/30 nm, 582/15 nm and 630/20 nm. Data were analyzed using the FlowJo software (Tree Star). This assay can be done in 96 or 384-well high-throughput format using 10 µl volume per sample, or it can be done in a standard format with higher volume. Cells were grown in RPMI-1640 containing 10% FBS, treated with drugs and labeled with fluorescent dyes for indicated times in a cell culture incubator. Cells were then sonicated with a single or 8-channel 2-mm probe for 3 x 1s pulses in the media in-well on ice, using an ultrasonicator (Sonics model VibraCell VCX130PB 130W 20 kHz from Sonics & Materials, Inc.) at 75% amplitude. Disruption of the cells could also be done with a 28 G 1/2 insulin syringe to generate equivalent results; however, sonication is easier, more reliable and reproducible than using a syringe. Cell homogenates are now ready to run on a flow cytometer at room temperature or 4°C if available. Optional: cells can be resuspended in FACS buffer (PBS containing 0.1–1%BSA and protease inhibitors (Sigma P-8340, 1∶2000 dilution)) on ice and labeled with fluorescent dyes after dislodging with trypsin/EDTA. For flow cytometry analysis, the events were first gated based on size (FSC) and granularity (SSC) and designated as “subcellular” population. The residual events detected in the subcellular population, defined by the FSC and SSC upon running the filtered buffer on a flow cytometry, possibly air-bubbles or impurities, represented <5% of total events and were not fluorescent. Then, specific AV population gate was created based on a specific fluorescent channel vs. the counter-stain or a non-specific channel, or on histogram. Number of AVs in this gate was counted and normalized to the number of cells. Cell numbers before sonication can be calculated by spiking in a known number of beads (CountBright counting beads, Invitrogen C36950, size 7 µm) and counting an aliquot of the mixture of cells and beads to deduce the cell numbers in each well proportionally, or by scanning on an IsoCyte (Molecular Devices) using cell areas as an approximation for cell numbers. In-sample cell number normalization can be done by splitting cell suspensions into two equal parts, then sonicating one part and combining it with the unsonicated half. Using this assay, many readout outputs may be calculated such as the number of organelles per cell, percentage of a specific population relative to a total number of events, or the total specific signal per cell based on the number of organelles per cell multiplied by the mean value of this parameter. This assay can work on either frozen or live cells, in growth media or in buffer. Cell lines successfully tried with this protocol include: PC3, LNCaP, HEK293, U87, MEF, 537MEL, BT474, SKBR3, ZR-75, MCF7, SKMEL23, MALME3, MALME3M and HME (American Type Culture Collection).

### Fluorescence Microscopy analysis

Cells stained with AO (0.1 µg/ml) for 30 min in a cell culture incubator were sonicated with 3 x 1-second pulses on ice in PBS containing 0.1% BSA and protease inhibitors (Sigma P-8340, 1∶2000 dilution) after dislodging with trypsin/EDTA. The cell homogenates were centrifuged in a 96 well plate in a Beckman Coulter “Allegra” X-12R centrifuge at 3000 rpm (2000 g) for 5 min and analyzed under a 100× objective on a DeltaVision microscope with an excitation setting for FITC and emission setting for Cy5. When bound to DNA, AO is fluorescent in the green FITC/FITC (ex 488 nm/em 530 nm) channel; when in acidic compartments, AO is fluorescent in the red FITC/Cy5 (ex 488 nm/em 650 nm) channel. In some studies, cells and organelles were analyzed under a 40× objective of a Nikon Eclipse TE300 microscope. mCherry was detected in an ex 561/em 600 channel. eGFP, LysoTrackerGreen, quinacrine, LynxTag-CQgreen were detected in the green FITC/FITC (ex 488 nm/em 530 nm) channel. LynxTag-CQblue and Hoechst 33342 were detected in the blue DAPI/DAPI (ex 405 nm/em 450 nm) channel.

### siRNA knockdown

Cells were transfected with 50 nM siRNA using Lipofectamine RNAiMAX. The On-Target-plus siControl Non-targeting pool (Dharmacon D-001810-10) was used as a non-targeting control. Atg5 siRNA pool (Dharmacon UNQ15221 siRNA IDs J-004374-07, J-004374-08, J-004374-09, J-004374-10) or Atg7 siRNA (Santa Cruz Biotech sc-41447) were used to specifically knockdown the Atg genes.

### Transient transfection of cells with plasmid DNA

Cells were transfected with plasmid DNA using Lipofectamine 2000 in 10 cm or 96-well tissue culture plates using 15 µg DNA and 15 µl Lipofectamine 2000 per plate in 10 ml growth media for 24–48 hours.

### Statistical analysis

Paired t-tests were performed using the Microsoft Excel software. Significant differences were determined as P<0.05.

## Supporting Information

Figure S1 **Microscopy images, western blot analysis and OFACS of PC3 cells treated with the indicated agents.** (**A,B**) PC3 cells treated for 2 days with 1 µM GDC-0941 and 10 µM CQ, stained with Hoechst 33342 and Acridine Orange (AO) and imaged with a GE InCell2000 microscope with a 20× objective. RGB images from blue, red and green channels are merged using Adobe Photoshop (**A**). Single channel and merged images of a cell treated with GDC-0941 and CQ are shown in (**B**). (**C**) PC3 cells treated for 24 hours with 1 µM GDC-0941 or 5 µM GDC-0068 +/− 10 µM CQ, stained with LysoTracker Red DND-99 and Hoechst 33342 and imaged with a 100× objective on a DeltaVision microscope. RGB images from red and blue channels are merged. (**D**) PC3 cells treated with 2 µM GDC-0941 +/− 10 µM CQ were analyzed by western blot analysis for LC3B and p62 at the indicated timepoints. (**E**) Cellular and subcellular populations of PC3 cells compared to different sizes of nano-beads by flow cytometry. Red gate: PC3 cells treated with 1 µM GDC-0941 and 10 µM chloroquine for 24 hours, then sonicated and analyzed by OFACS. Blue gate: 7 µm beads (Count-bright beads, Invitrogen C36950). Green gate: Non-fluorescent 90 nm (50–100 nm) beads (Spherotech PP-008-10). Beads were diluted 1∶10 in PBS/0.2% Triton X-100, sonicated 5 s, then ran on flow cytometer. FSC histogram (left) shows approximate size distribution: the subcellular population has similar FSC value to the 90 nm beads, and 7 µm beads have the FSC value intermediate between subcellular and cellular populations. FSC/SSC plots (right) show that the subcellular population has similar FSC/SSC profile to the 90 nm beads. Individual histograms and dot-plots were overlaid in the bottom panels. (**F**) PC3 cells treated with 1 µM GDC-0941 +/− 10 µM CQ for 24 hours were sonicated and mixed with unsonicated parts of the sample at 1∶1 ratio. The sonicated part represents the subcellular population, and the unsonicated part represents the cellular population. Having unbroken (unsonicated) cells together with the sonicated material gives an advantage of having the internal control for the number of cells, present in each individual sample. The number of events in the subcellular population is divided by the number of events in the cellular population to obtain the normalized subcellular events. Scale bars, 20 µm.
(TIF)

Figure S2 **Microscopy images of parental and mCherry-eGFP-LC3B expressing PC3 cells.** (**A**) PC3 cells treated for 2 days with 1 µM GDC-0941 and 10 µM CQ, stained with LysoTracker Green DND-26 and Hoechst 33342, sonicated, pelleted and imaged with a 100× objective on a DeltaVision microscope. Left, bottom focus plane: released vacuoles on the bottom of the plate are in focus. Right, mid-cell level focus: vacuoles within an unbroken cell in focus, free vacuoles on the bottom of the plate are out of focus. (**B**–**C**) PC3 cells stably expressing mCherry-eGFP-LC3B were treated with 5 µM GDC-0941 (**B**) or GDC-0068 (**C**) +/− 10 µM CQ for 24 hours and imaged under microscope with a 40× objective. mCherry (red) and eGFP (green) channels are merged. Scale bars, 10 µm (**A**) and 20 µm (**B & C**).
(TIF)

Figure S3 **Western blot analysis of knockdown efficiency by Atg5 and Atg7 siRNAs.** (**A**) ATG5 and ATG7 immunoblots in Wild-type (WT) PC3 cells or PC3 cells stably expressing mCherry-eGFP-LC3B transfected with non-targeting (NT) siRNA or siRNAs against Atg5 or Atg7. Cells were lysed 2 days after transfection and analyzed with with ATG5, ATG7 or GAPDH antibodies. (**B**) Quantification of Atg5 and Atg7 protein levels in (**A**) on a LiCOR Odyssey system.
(TIF)

Figure S4 **Comparison of OFACS readout outputs.** (**A**–**E**) PC3 cells treated for 2 days with 1 µM GDC-0941 or 5 µM GDC-0068 +/− 10 µM CQ, stained with AO, sonicated, and AO^+^ organelles analyzed by OFACS showing the related outputs: (**A**) normalized total number of all subcellular events; (**B**) number of AO^+^ organelles per cell; (**C**) normalized number of LysoTrackerRed^+^ events; (**D**) normalized total red signal intensity of AO^+^ events; (**E**) percentage of AO^+^ events of all events. Error bars represent standard errors of more than 3 experiments. (**F**–**H**) HEK293 cells treated for 2 days with 1 µM GDC-0941 +/− 10 µM CQ, stained with LysoTrackerRed DND-99 or AO, sonicated, and analyzed by OFACS. (**F**) Normalized total number of subcellular events. (**G**) Normalized number of AO^+^ events. (**H**) Normalized number of LysoTrackerRed^+^ events. Error bars represent standard errors of 3 experiments. (**I**) Time course of the accumulation of AO^+^ organelles. Same data were plotted on different y-axis scales on the left and the right panels. PC3 cells were treated with 1 µM GDC-0941 +/− 10 µM CQ for different periods of time, stained with AO and analyzed by OFACS after sonication. Starting at about 3–6 hours, AO^+^ organelles accumulated over time with dual drug treatment showing the strongest accumulation, as indicated by the number of AO^+^ organelles per cell. Error bars represent standard errors of 4 experiments. (**J**–**K**) AV accumulation as a function of CQ concentration. PC3 cells were treated for 2 days with 1 µM GDC-0941 with increasing concentrations of CQ, stained with AO and analyzed by OFACS after sonication. Two different outputs, the normalized number of AO^+^ events (**J**) and the percentage of AO^+^ events of total subcellular population (**K**), are shown. Error bars represent SEM (n = 4).
(TIF)

Figure S5 **Concentration-dependent accumulation of AO^+^ organelles at different combinations of GDC-0941 and CQ analyzed by OFACS in a 96-well format.** PC3 cells were treated for 2 days with combinations of GDC-0941 (rows) and CQ (columns) at varying concentrations in a 96 well format, stained with AO and analyzed by OFACS. (**A**) 96-well plate layout of drug concentrations and corresponding percentage of AO^+^ events. Numbers are color-coded according to the degree of accumulation. Green: 0–20%; yellow: 20–70%; red: 70–100%. (**B**) Bliss independence analysis of data in (**A**) showing deviation of the experimental data from Bliss independence at each concentration pair of GDC-0941 and CQ. Bliss independence (fraction, 0 to1)  =  (drug A effect value) + (drug B effect value) - (drug A effect value) x (drug B effect value). The higher the score the stronger the synergistic effect. The Bliss deviation is color-coded as Green: 0–10%; yellow: 10–30%; red: 30–100%. (**C)** and (**D**): data from (**A**) and (**B**) graphed in 3-D, respectively.
(TIF)

Figure S6 **OFACS analysis of eGFP-LC3B expressing PC3 cells treated with GDC-0941 +/− CQ.** PC3 cells stably expressing eGFP-LC3B were treated with GDC-0941 (3 µM) +/− CQ (10 µM) for 24 hours, stained with Hoechst 33342 and analyzed by OFACS after sonication. (**A**) Flow cytometry plots of eGFP (FITC channel) vs. counter-stain Hoechst (DAPI channel) of the “subcellular” population. A distinct GFP-positive population is circled with the corresponding percentage of total events in the subcellular population. (**B**) Corresponding histograms of the “subcellular” population for eGFP intensity (FITC channel). (**C,D**) Free mCherry protein co-localized with eGFP-LC3B in PC3 cells treated with GDC-0941 or GDC-0068 and protease inhibitors or CQ by OFACS assay. PC3 cells stably expressing eGFP-LC3B were transiently transfected with mCherry for 48 hours, then treated with GDC-0941 (1 µM) or GDC-0068 (5 µM) +/−CQ (10 µM) with and without a protease inhibitor cocktail P1860 or 3-MA (3 mM) for another 48 hours, then sonicated and analyzed by OFACS. (**C**) Normalized number of eGFP^+^ events in the FITC channel. (**D**) Normalized number of eGFP^+^mCherry^+^ events detected in both FITC and PE-Texas Red channels. Error bars represent SEM (n = 4). *, P<0.05 vs. DMSO group with the same autophagy inhibitors. NS, non-significant (P>0.05). (**E**) Graphic representation of the comparison between microscopy (MS) and OFACS (OFACS) quantifications of the different treatments shown in Table S1. Error bars represent standard errors from 3 experiments.
(TIF)

Figure S7 **Image and OFACS analysis of PC3 cells stably expressing LC3B-RFP or LC3B-G120A-RFP.** PC3 cells were transduced with Premo™ Autophagy Sensor LC3B-RFP (BacMam 2.0) kit (Invitrogen P36236) and sorted by flow cytometer for RFP-positive cells. Cells were treated with 1 uM GDC-0941 +/− 10 uM CQ for 24 hours in 96-well plates, then stained with 1 uM quinacrine for 45 minutes in incubator. Cells were imaged live with a Nikon Eclipse TE300 microscope with a 40× objective. RFP was detected in (550 nm ex/590 nm em) channel, quinacrine was detected in (488 nm ex/530 nm em) channel. After imaging, the same samples were analyzed by OFACS. RFP was detected in the dTomato channel(561 nm ex/582 nm em), quinacrine was detected in the FITC channel. Scale bar, 50 µm. (**A**) Fluorescent microscopy showing co-localization of RFP and quinacrine stained dots for LC3B but not for LC3B-G120A mutant. LC3B-G120A-RFP mutant failed to form puncta after autophagy induction with GDC-0941 and inhibition with CQ. (**B**) RFP histograms of sub-cellular populations from OFACS analysis. Black: LC3B-RFP DMSO; Red: LC3B-RFP GDC-0941 +CQ; Blue: LC3B-G120A-RFP DMSO; Green: LC3B-G120A-RFP GDC-0941 +CQ. Dotted line represents an arbitrary boundary for RFP+ events. (**C**) OFACS analysis of RFP vs. Quinacrine dot plots showing drug treatment-dependent increase of events in RFP^+^QC^+^ quadrant for LC3B but not for LC3B-G120A mutant. (**D**) OFACS quantitation from (**B**): normalized number of RFP^+^QC^+^ subcellular events. * P<0.05. Error bars represent SEM (n = 3).
(TIF)

Figure S8 **PC3 cells treated with HBSS or rapamycin and analyzed by OFACS.** (**A**) Normalized GFP^+^ events at the timepoints indicated. PC3 cells stably expressing eGFP-LC3B were grown in full media or starved with HBSS and treated with or without 10 µM CQ for 8 and 24 hours. (**B**) Representative images of GFP-LC3B^+^ dots of cells in (**A**) was confirmed by imaging microscopy with a 40× objective. Scale bar, 20 µm. (**C**) PC3 cells treated with indicated concentrations of GDC-0941 or Rapamycin with or without 10 uM CQ for 24 hours were stained with AO and analyzed by OFACS. Normalized number of AO^+^ events are shown. Error bars represent SEM (n = 3).
(TIF)

Figure S9 **PC3 cells expressing mCherry-eGFP-LC3B are analyzed by OFACS using Image flow cytometry and compared to conventional flow cytometry.** Representative images of cells (**A**) and mCherry^+^GFP^+^ AVs (**B**) analyzed by imaging flow cytometry analysis on an ImageStream cytometer. (**C**) Gates used to define single cells and organelles and their doublets with the ImageStream analysis. Brightfield area is shown on the x-axis, and brightfield aspect ratio is shown on the y-axis. Statistics of different populations are shown in the table under the plot. Sonicated cell homogenates were mixed with unsonicated homogenates to show the position of intact cells and cell doublets. (**D**) Numbers of mCherry^+^eGFP^+^ AVs obtained by the ImageStream analysis compared to those obtained using conventional flow cytometry. Data are represented as Mean ± SEM (n = 3). PC3 cells expressing mCherry-eGFP-LC3B were treated with 2 µM GDC-0941 +/− 10 µM CQ for 24 hours. AVs were analyzed by OFACS from aliquots of the same samples using conventional flow cytometer and compared to the image flow cytometry analysis on an ImageStream cytometer (Amnis Corporation). Fluorescent signals were determined as for Fluorescence Microscopy analysis: GFP in ex 488 nm/em 530 nm channel and mCherry in ex 561 nm/em 600 nm channel.
(TIF)

Figure S10 **Fluorescent labeling of autophagic compartments.** (**A**) LynxTagCQ-blue labels acidic vesicles in PC3 cells with a blue fluorescence. PC3 cells were treated with LynxTagCQ-blue (5 µM) +/− GDC-0941 (1 µM) for 24 hours and imaged with a 40× objective under microscope in the blue (DAPI) channel. (**B**) Labeling of AVs by eGFP-p62 in PC3 cells. PC3 cells were transfected with eGFP-p62 for 24 hours, then treated with GDC-0941 (1 µM) +/− CQ (10 µM) for 24 hours, stained with Hoechst 33342 and imaged with a 40× objective under microscope in green (FITC) and blue (DAPI) channels. Merged images in green and blue channels are shown. (**C,D**) Quinacrine-labeled AVs are fluorescent in the green channel and can be detected by OFACS. PC3 cells were treated with GDC-0941 (1 µM) +/− CQ (10 µM) for 24 hours, then stained with 1 µM quinacrine for 1 hour and imaged with a 40× objective under microscope in a green (FITC) channel (**C**). The same samples were then sonicated and analyzed by OFACS (**D**). Scale bars, 20 µm. (**E**) Dose response of CQ (AO^+^) and QC (FITC^+^) in inducing stained subcellular events by OFACS analysis. Error bars represent SEM (n = 3).
(TIF)

Figure S11 **Co-localization of LynxTagCQ-blue labeled organelles with Acridine Orange.** PC3 cells were treated +/− GDC-0941 (1 µM) with increasing concentrations of LynxTagCQ-blue (0–5 µM) for 24 hours, stained with AO, then sonicated and analyzed by OFACS. (**A**) Histograms in the blue (DAPI) channel. (**B**) Corresponding red (PerCP) vs. blue (DAPI) channel dot plots of the “organelle” population.
(TIF)

Figure S12 **Contribution of subcellular membranes to the subcellular population in PC3 homogenates.** (**A**–**D**) Representative images of mitochondria labeled with CellLight-Mitochondria-RFP kit (Invitrogen C10601) according to the manufacturer's protocol for 24 hours. Then cells were treated with 1 uM GDC-0941 +/− 10 uM CQ for 24 hours, then stained with 1 uM quinacrine for 45 minutes. Cells were imaged live with a 40× objective. Scale bars, 50 µm. RFP was detected in (550 nm ex/590 nm em) channel, quinacrine was detected in (488 nm ex/530 nm em) channel. After cell imaging, the same samples were analyzed by OFACS. Homogenates were centrifuged at 2000 g for 10 minutes in a glass bottom 96-well plate and imaged with a 100× objective using the same channel filters. RFP-labeled mitochondria (red arrow) and quinacrine-stained AVs (green arrow) are indicated in DMSO (Upper panel) and GDC-091+CQ (lower panel) treated samples. (**E**) Quantification of % marker positive events labeled with CellLight kits expressing the indicated organelle markers and QC. Numbers of RFP^+^QC^−^event were normalized to the number of RFP^+^ cells and calculated as the percentage of the normalized total subcellular events. Error bars represent SEM (n = 3).
(TIF)

Figure S13 **Flow cytometry sorting of AVs labeled with three different fluorophores.** PC3 cells expressing mCherry-eGFP-LC3B were treated with 2 µM GDC-0941, 9 µM CQ and 1 µM LynxTag-CQ-blue (CQblue) for 24 hours, then sonicated according to the OFACS protocol. AVs labeled with three different fluorophores (mCherry, eGFP, LynxTag-CQblue) were subjected to flow cytometry sorting on a FacsAria flow cytometer sorter. CQ-blue was detected in PacificBlue channel (ex 405 nm/em 455 nm). Specific AVs (**A**) and debris (**B**) populations were established by backgating analysis and used as such for sorting. 2000 events from unsorted (**C**) and sorted (**D**) samples are shown. Populations for “organelles”, double fluorophore PacificBlue^+^mCherry^+^ or triple fluorophore PacificBlue^+^mCherry^+^GFP^+^ populations are circled with corresponding percentage in each population.
(TIF)

Materials and Methods S1 **Supplementary Materials and Methods.**
(DOCX)

Table S1 **Comparison of number of AVs per cell obtained by microscopic analysis and by OFACS analysis.**
(PDF)
