# Supplementary figures and images for: Novel Quantitative Autophagy Analysis by Organelle Flow Cytometry after Cell Sonication
Source: PLoS One. 2014 Jan 29;9(1):e87707. doi: 10.1371/journal.pone.0087707 (PMC3906200; doi:10.1371/journal.pone.0087707)

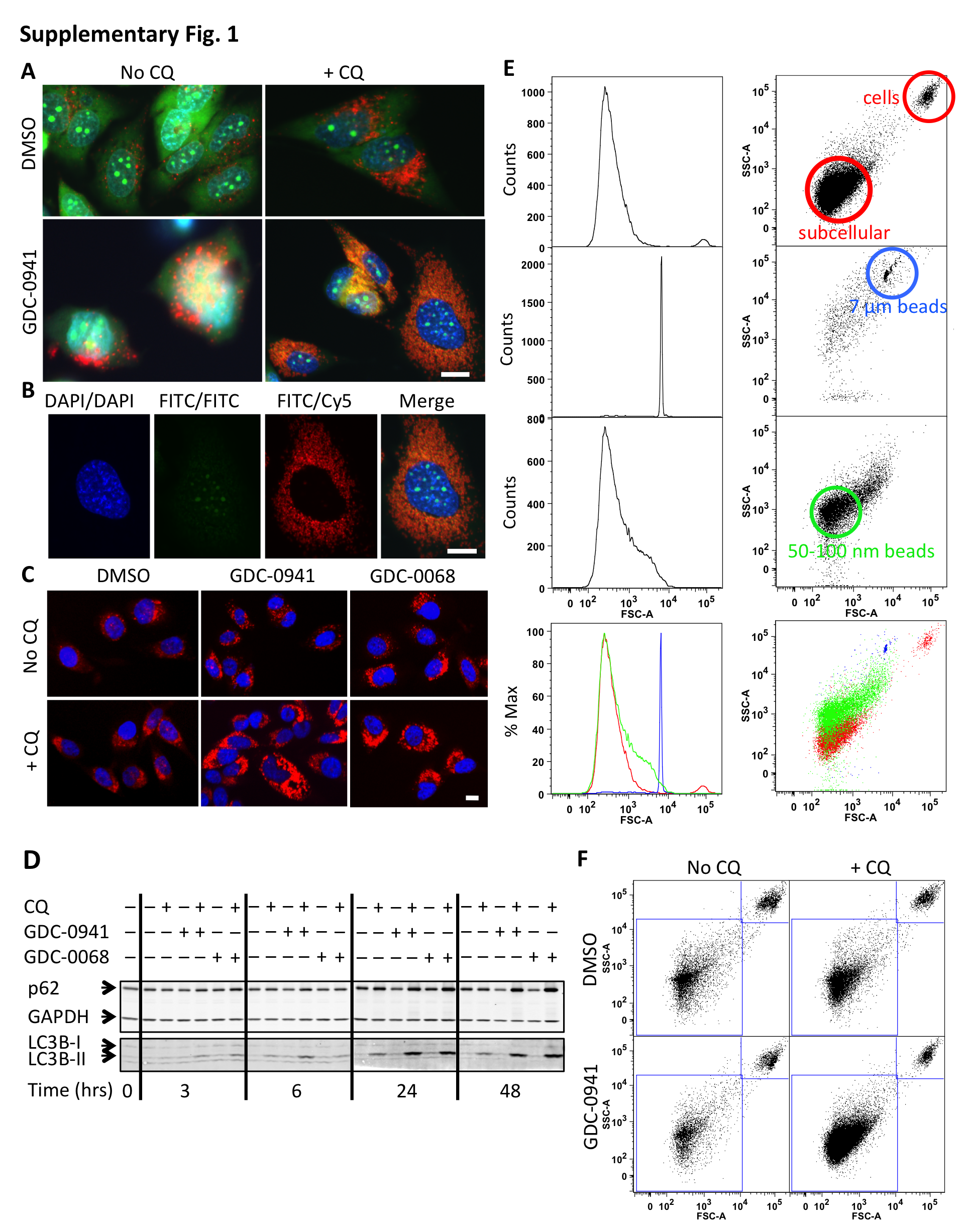

Supplement: Figure S1 — Microscopy images, western blot analysis and OFACS of PC3 cells treated with the indicated agents. (A,B) PC3 cells treated for 2 days with 1 µM GDC-0941 and 10 µM CQ, stained with Hoechst 33342 and Acridine Orange (AO) and imaged with a GE InCell2000 microscope with a 20× objective. RGB images from blue, red and green channels are merged using Adobe Photoshop (A). Single channel and merged images of a cell treated with GDC-0941 and CQ are shown in (B). (C) PC3 cells treated for 24 hours with 1 µM GDC-0941 or 5 µM GDC-0068 +/− 10 µM CQ, stained with LysoTracker Red DND-99 and Hoechst 33342 and imaged with a 100× objective on a DeltaVision microscope. RGB images from red and blue channels are merged. (D) PC3 cells treated with 2 µM GDC-0941 +/− 10 µM CQ were analyzed by western blot analysis for LC3B and p62 at the indicated timepoints. (E) Cellular and subcellular populations of PC3 cells compared to different sizes of nano-beads by flow cytometry. Red gate: PC3 cells treated with 1 µM GDC-0941 and 10 µM chloroquine for 24 hours, then sonicated and analyzed by OFACS. Blue gate: 7 µm beads (Count-bright beads, Invitrogen C36950). Green gate: Non-fluorescent 90 nm (50–100 nm) beads (Spherotech PP-008-10). Beads were diluted 1∶10 in PBS/0.2% Triton X-100, sonicated 5 s, then ran on flow cytometer. FSC histogram (left) shows approximate size distribution: the subcellular population has similar FSC value to the 90 nm beads, and 7 µm beads have the FSC value intermediate between subcellular and cellular populations. FSC/SSC plots (right) show that the subcellular population has similar FSC/SSC profile to the 90 nm beads. Individual histograms and dot-plots were overlaid in the bottom panels. (F) PC3 cells treated with 1 µM GDC-0941 +/− 10 µM CQ for 24 hours were sonicated and mixed with unsonicated parts of the sample at 1∶1 ratio. The sonicated part represents the subcellular population, and the unsonicated part represents the cellular population. Having unbroken (unsonic [file pone.0087707.s001.tif]

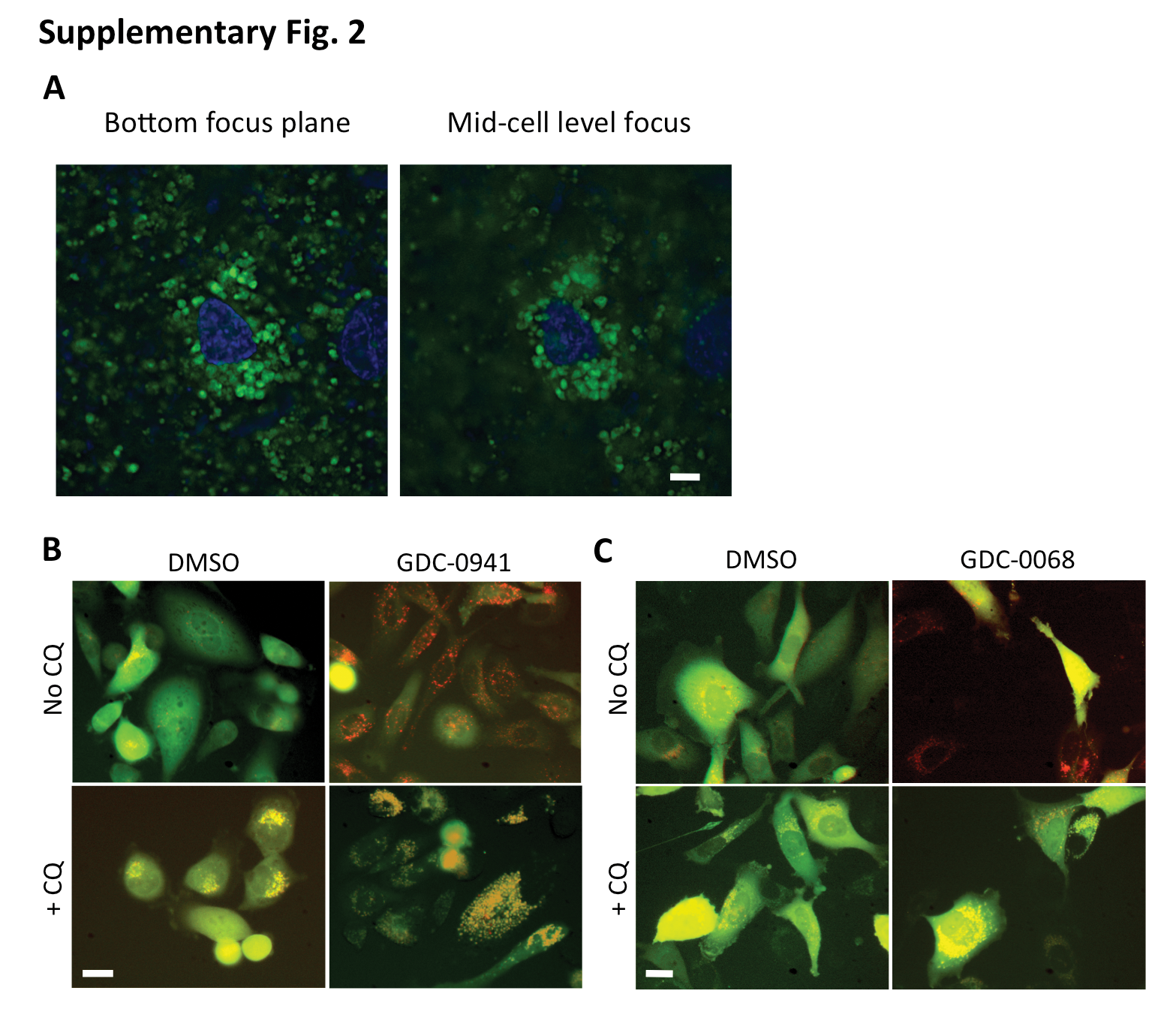

Supplement: Figure S2 — Microscopy images of parental and mCherry-eGFP-LC3B expressing PC3 cells. (A) PC3 cells treated for 2 days with 1 µM GDC-0941 and 10 µM CQ, stained with LysoTracker Green DND-26 and Hoechst 33342, sonicated, pelleted and imaged with a 100× objective on a DeltaVision microscope. Left, bottom focus plane: released vacuoles on the bottom of the plate are in focus. Right, mid-cell level focus: vacuoles within an unbroken cell in focus, free vacuoles on the bottom of the plate are out of focus. (B–C) PC3 cells stably expressing mCherry-eGFP-LC3B were treated with 5 µM GDC-0941 (B) or GDC-0068 (C) +/− 10 µM CQ for 24 hours and imaged under microscope with a 40× objective. mCherry (red) and eGFP (green) channels are merged. Scale bars, 10 µm (A) and 20 µm (B & C). (TIF) [file pone.0087707.s002.tif]

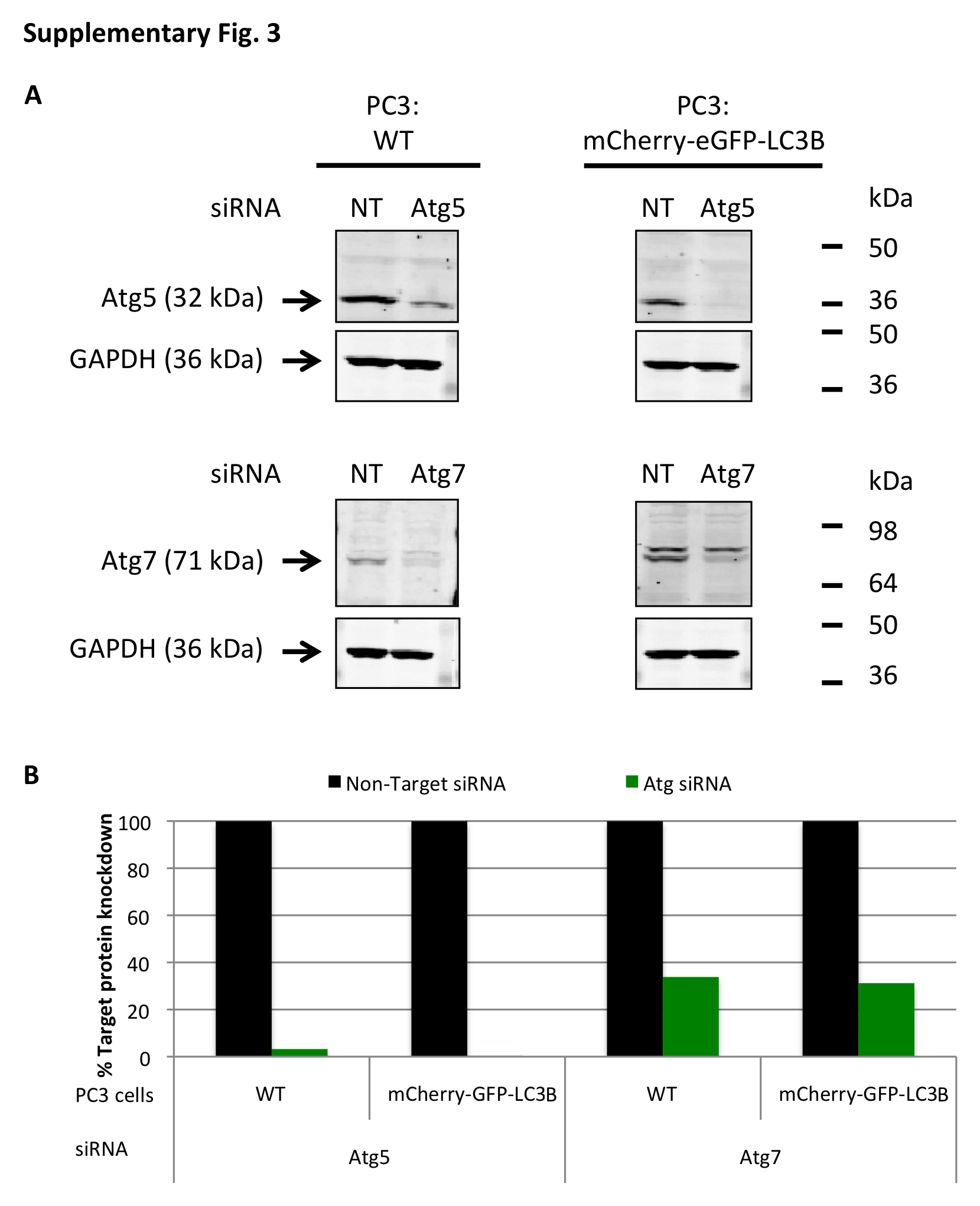

Supplement: Figure S3 — Western blot analysis of knockdown efficiency by Atg5 and Atg7 siRNAs. (A) ATG5 and ATG7 immunoblots in Wild-type (WT) PC3 cells or PC3 cells stably expressing mCherry-eGFP-LC3B transfected with non-targeting (NT) siRNA or siRNAs against Atg5 or Atg7. Cells were lysed 2 days after transfection and analyzed with with ATG5, ATG7 or GAPDH antibodies. (B) Quantification of Atg5 and Atg7 protein levels in (A) on a LiCOR Odyssey system. (TIF) [file pone.0087707.s003.tif]

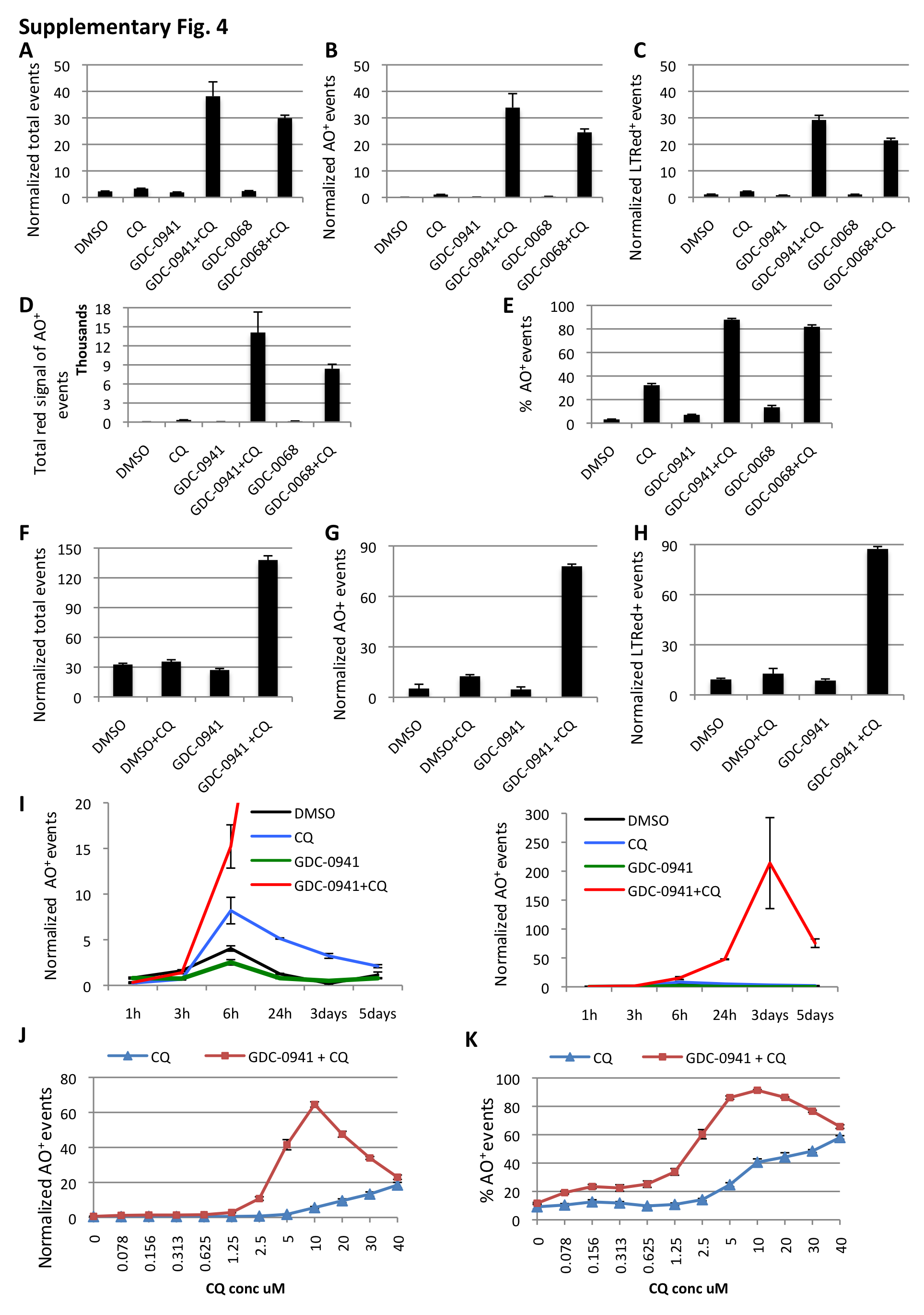

Supplement: Figure S4 — Comparison of OFACS readout outputs. (A–E) PC3 cells treated for 2 days with 1 µM GDC-0941 or 5 µM GDC-0068 +/− 10 µM CQ, stained with AO, sonicated, and AO+ organelles analyzed by OFACS showing the related outputs: (A) normalized total number of all subcellular events; (B) number of AO+ organelles per cell; (C) normalized number of LysoTrackerRed+ events; (D) normalized total red signal intensity of AO+ events; (E) percentage of AO+ events of all events. Error bars represent standard errors of more than 3 experiments. (F–H) HEK293 cells treated for 2 days with 1 µM GDC-0941 +/− 10 µM CQ, stained with LysoTrackerRed DND-99 or AO, sonicated, and analyzed by OFACS. (F) Normalized total number of subcellular events. (G) Normalized number of AO+ events. (H) Normalized number of LysoTrackerRed+ events. Error bars represent standard errors of 3 experiments. (I) Time course of the accumulation of AO+ organelles. Same data were plotted on different y-axis scales on the left and the right panels. PC3 cells were treated with 1 µM GDC-0941 +/− 10 µM CQ for different periods of time, stained with AO and analyzed by OFACS after sonication. Starting at about 3–6 hours, AO+ organelles accumulated over time with dual drug treatment showing the strongest accumulation, as indicated by the number of AO+ organelles per cell. Error bars represent standard errors of 4 experiments. (J–K) AV accumulation as a function of CQ concentration. PC3 cells were treated for 2 days with 1 µM GDC-0941 with increasing concentrations of CQ, stained with AO and analyzed by OFACS after sonication. Two different outputs, the normalized number of AO+ events (J) and the percentage of AO+ events of total subcellular population (K), are shown. Error bars represent SEM (n = 4). (TIF) [file pone.0087707.s004.tif]

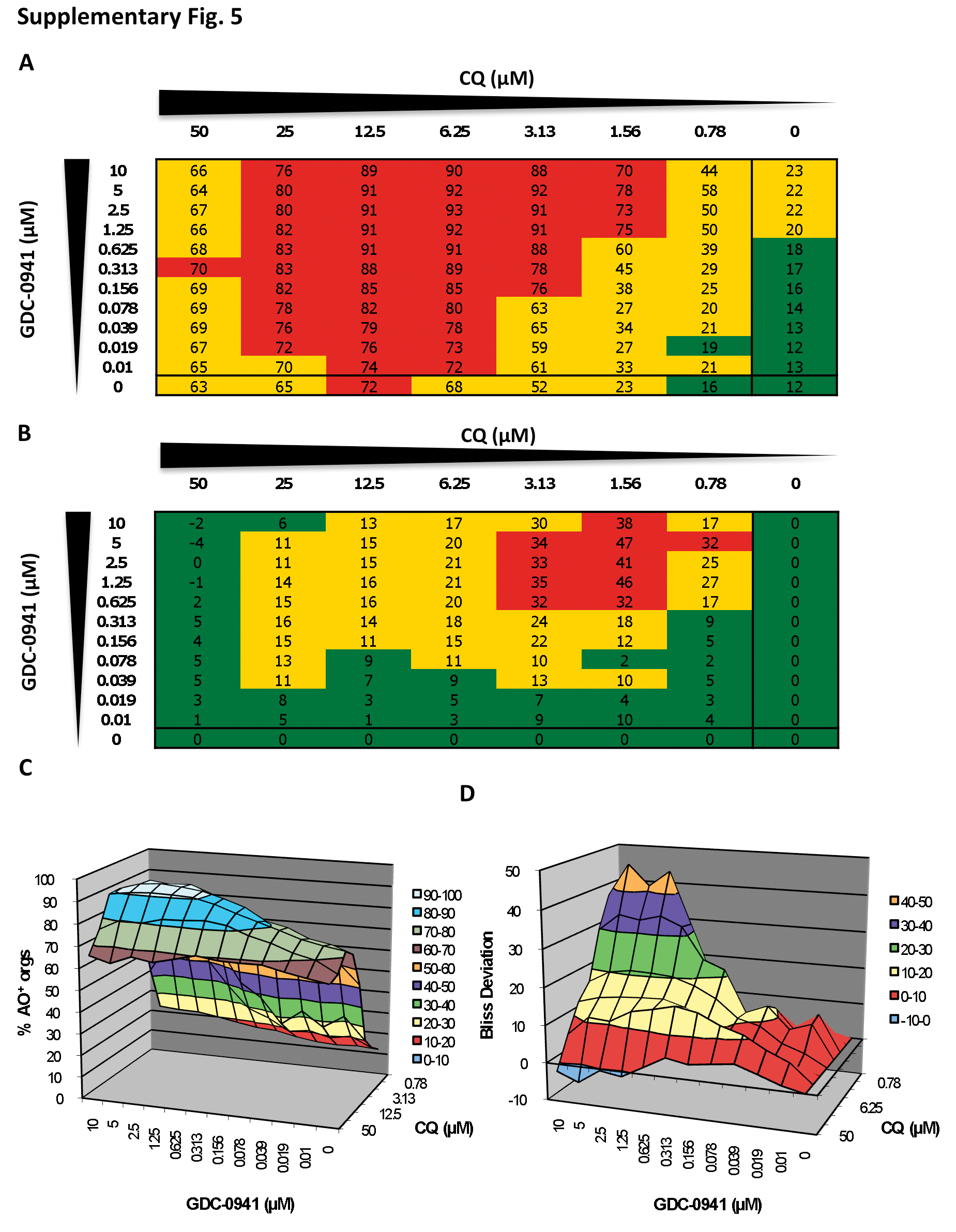

Supplement: Figure S5 — Concentration-dependent accumulation of AO+ organelles at different combinations of GDC-0941 and CQ analyzed by OFACS in a 96-well format. PC3 cells were treated for 2 days with combinations of GDC-0941 (rows) and CQ (columns) at varying concentrations in a 96 well format, stained with AO and analyzed by OFACS. (A) 96-well plate layout of drug concentrations and corresponding percentage of AO+ events. Numbers are color-coded according to the degree of accumulation. Green: 0–20%; yellow: 20–70%; red: 70–100%. (B) Bliss independence analysis of data in (A) showing deviation of the experimental data from Bliss independence at each concentration pair of GDC-0941 and CQ. Bliss independence (fraction, 0 to1) = (drug A effect value) + (drug B effect value) - (drug A effect value) x (drug B effect value). The higher the score the stronger the synergistic effect. The Bliss deviation is color-coded as Green: 0–10%; yellow: 10–30%; red: 30–100%. (C) and (D): data from (A) and (B) graphed in 3-D, respectively. (TIF) [file pone.0087707.s005.tif]

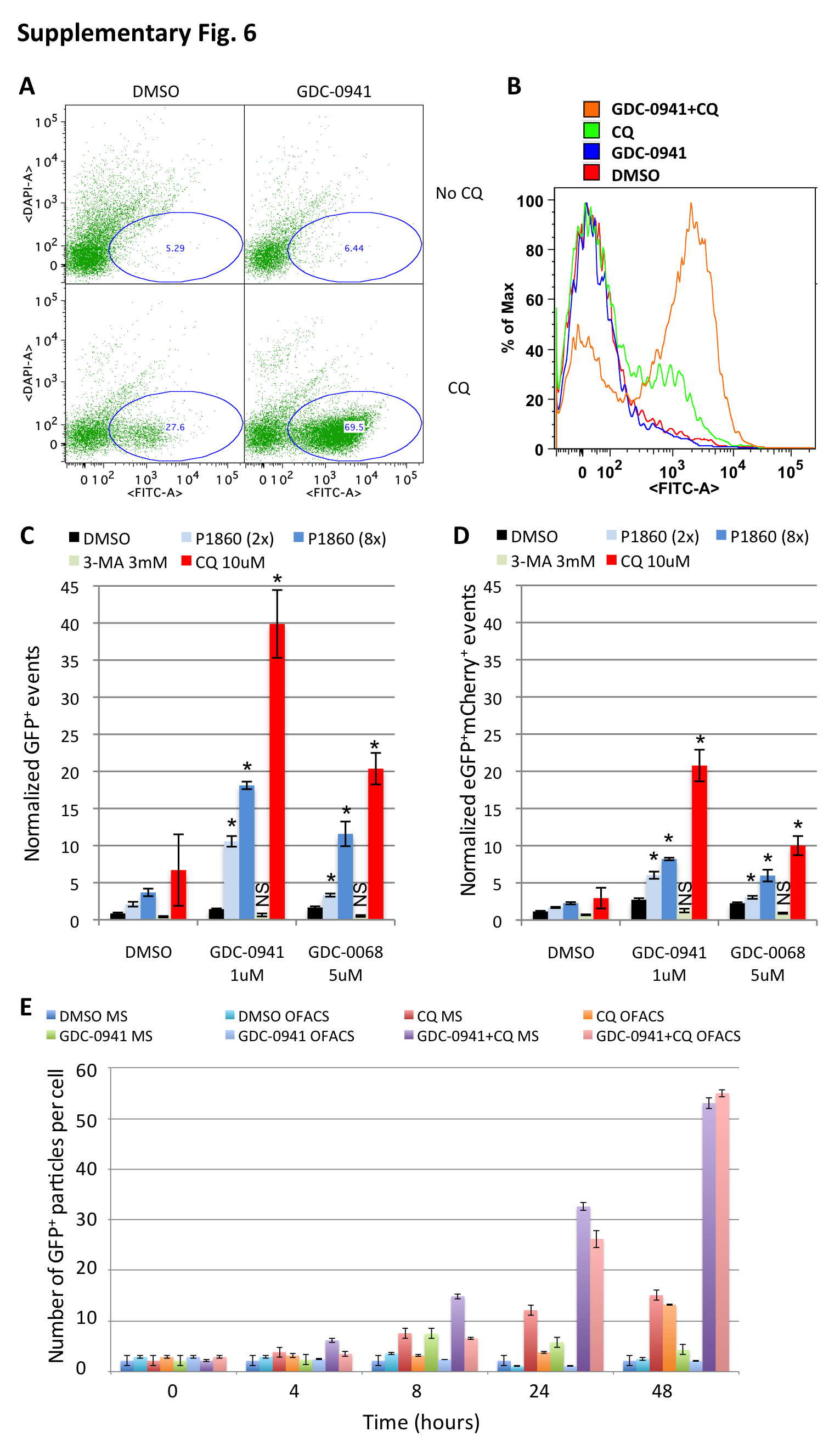

Supplement: Figure S6 — OFACS analysis of eGFP-LC3B expressing PC3 cells treated with GDC-0941 +/− CQ. PC3 cells stably expressing eGFP-LC3B were treated with GDC-0941 (3 µM) +/− CQ (10 µM) for 24 hours, stained with Hoechst 33342 and analyzed by OFACS after sonication. (A) Flow cytometry plots of eGFP (FITC channel) vs. counter-stain Hoechst (DAPI channel) of the “subcellular” population. A distinct GFP-positive population is circled with the corresponding percentage of total events in the subcellular population. (B) Corresponding histograms of the “subcellular” population for eGFP intensity (FITC channel). (C,D) Free mCherry protein co-localized with eGFP-LC3B in PC3 cells treated with GDC-0941 or GDC-0068 and protease inhibitors or CQ by OFACS assay. PC3 cells stably expressing eGFP-LC3B were transiently transfected with mCherry for 48 hours, then treated with GDC-0941 (1 µM) or GDC-0068 (5 µM) +/−CQ (10 µM) with and without a protease inhibitor cocktail P1860 or 3-MA (3 mM) for another 48 hours, then sonicated and analyzed by OFACS. (C) Normalized number of eGFP+ events in the FITC channel. (D) Normalized number of eGFP+mCherry+ events detected in both FITC and PE-Texas Red channels. Error bars represent SEM (n = 4). *, P<0.05 vs. DMSO group with the same autophagy inhibitors. NS, non-significant (P>0.05). (E) Graphic representation of the comparison between microscopy (MS) and OFACS (OFACS) quantifications of the different treatments shown in Table S1. Error bars represent standard errors from 3 experiments. (TIF) [file pone.0087707.s006.tif]

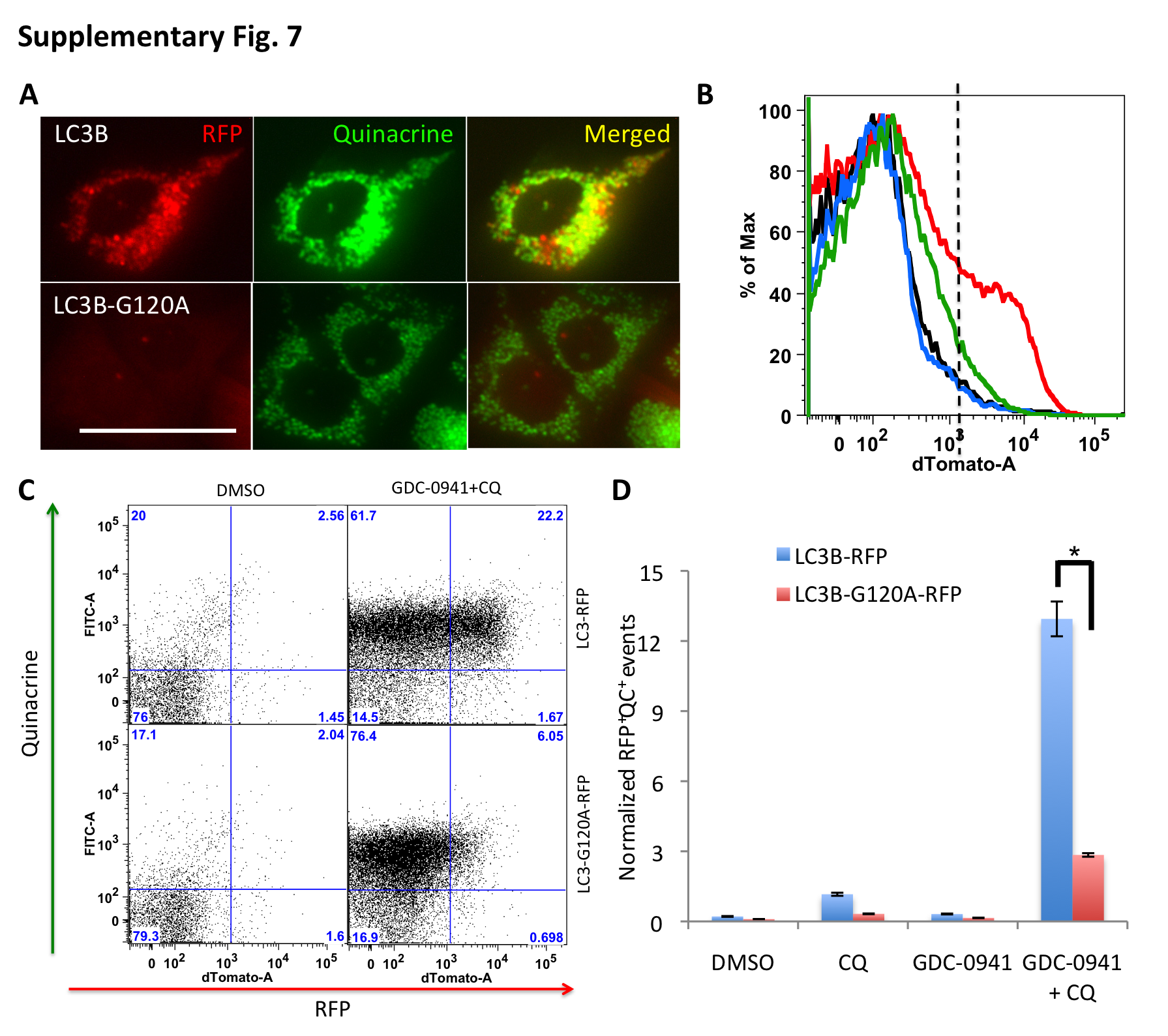

Supplement: Figure S7 — Image and OFACS analysis of PC3 cells stably expressing LC3B-RFP or LC3B-G120A-RFP. PC3 cells were transduced with Premo™ Autophagy Sensor LC3B-RFP (BacMam 2.0) kit (Invitrogen P36236) and sorted by flow cytometer for RFP-positive cells. Cells were treated with 1 uM GDC-0941 +/− 10 uM CQ for 24 hours in 96-well plates, then stained with 1 uM quinacrine for 45 minutes in incubator. Cells were imaged live with a Nikon Eclipse TE300 microscope with a 40× objective. RFP was detected in (550 nm ex/590 nm em) channel, quinacrine was detected in (488 nm ex/530 nm em) channel. After imaging, the same samples were analyzed by OFACS. RFP was detected in the dTomato channel(561 nm ex/582 nm em), quinacrine was detected in the FITC channel. Scale bar, 50 µm. (A) Fluorescent microscopy showing co-localization of RFP and quinacrine stained dots for LC3B but not for LC3B-G120A mutant. LC3B-G120A-RFP mutant failed to form puncta after autophagy induction with GDC-0941 and inhibition with CQ. (B) RFP histograms of sub-cellular populations from OFACS analysis. Black: LC3B-RFP DMSO; Red: LC3B-RFP GDC-0941 +CQ; Blue: LC3B-G120A-RFP DMSO; Green: LC3B-G120A-RFP GDC-0941 +CQ. Dotted line represents an arbitrary boundary for RFP+ events. (C) OFACS analysis of RFP vs. Quinacrine dot plots showing drug treatment-dependent increase of events in RFP+QC+ quadrant for LC3B but not for LC3B-G120A mutant. (D) OFACS quantitation from (B): normalized number of RFP+QC+ subcellular events. * P<0.05. Error bars represent SEM (n = 3). (TIF) [file pone.0087707.s007.tif]

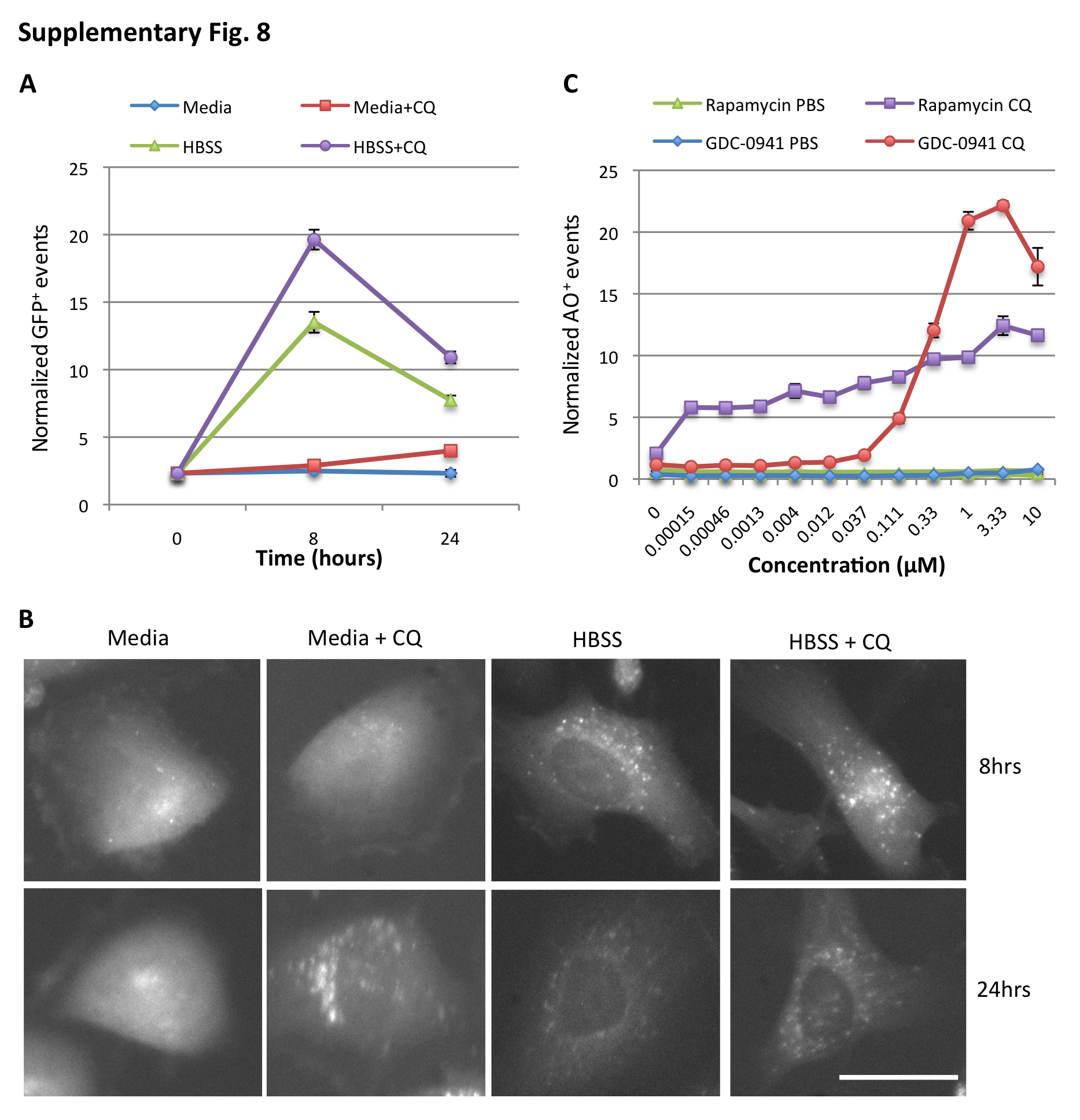

Supplement: Figure S8 — PC3 cells treated with HBSS or rapamycin and analyzed by OFACS. (A) Normalized GFP+ events at the timepoints indicated. PC3 cells stably expressing eGFP-LC3B were grown in full media or starved with HBSS and treated with or without 10 µM CQ for 8 and 24 hours. (B) Representative images of GFP-LC3B+ dots of cells in (A) was confirmed by imaging microscopy with a 40× objective. Scale bar, 20 µm. (C) PC3 cells treated with indicated concentrations of GDC-0941 or Rapamycin with or without 10 uM CQ for 24 hours were stained with AO and analyzed by OFACS. Normalized number of AO+ events are shown. Error bars represent SEM (n = 3). (TIF) [file pone.0087707.s008.tif]

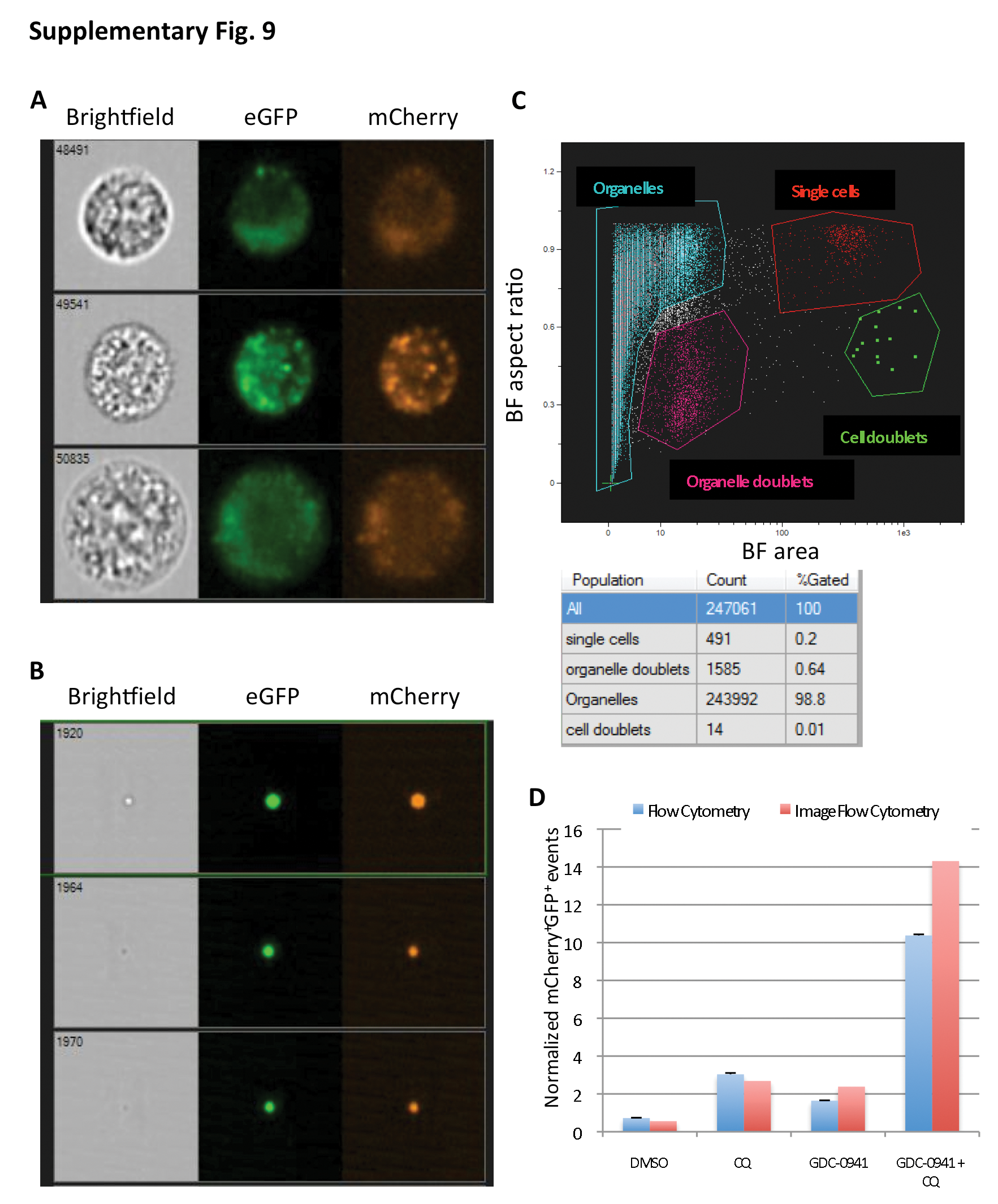

Supplement: Figure S9 — PC3 cells expressing mCherry-eGFP-LC3B are analyzed by OFACS using Image flow cytometry and compared to conventional flow cytometry. Representative images of cells (A) and mCherry+GFP+ AVs (B) analyzed by imaging flow cytometry analysis on an ImageStream cytometer. (C) Gates used to define single cells and organelles and their doublets with the ImageStream analysis. Brightfield area is shown on the x-axis, and brightfield aspect ratio is shown on the y-axis. Statistics of different populations are shown in the table under the plot. Sonicated cell homogenates were mixed with unsonicated homogenates to show the position of intact cells and cell doublets. (D) Numbers of mCherry+eGFP+ AVs obtained by the ImageStream analysis compared to those obtained using conventional flow cytometry. Data are represented as Mean ± SEM (n = 3). PC3 cells expressing mCherry-eGFP-LC3B were treated with 2 µM GDC-0941 +/− 10 µM CQ for 24 hours. AVs were analyzed by OFACS from aliquots of the same samples using conventional flow cytometer and compared to the image flow cytometry analysis on an ImageStream cytometer (Amnis Corporation). Fluorescent signals were determined as for Fluorescence Microscopy analysis: GFP in ex 488 nm/em 530 nm channel and mCherry in ex 561 nm/em 600 nm channel. (TIF) [file pone.0087707.s009.tif]

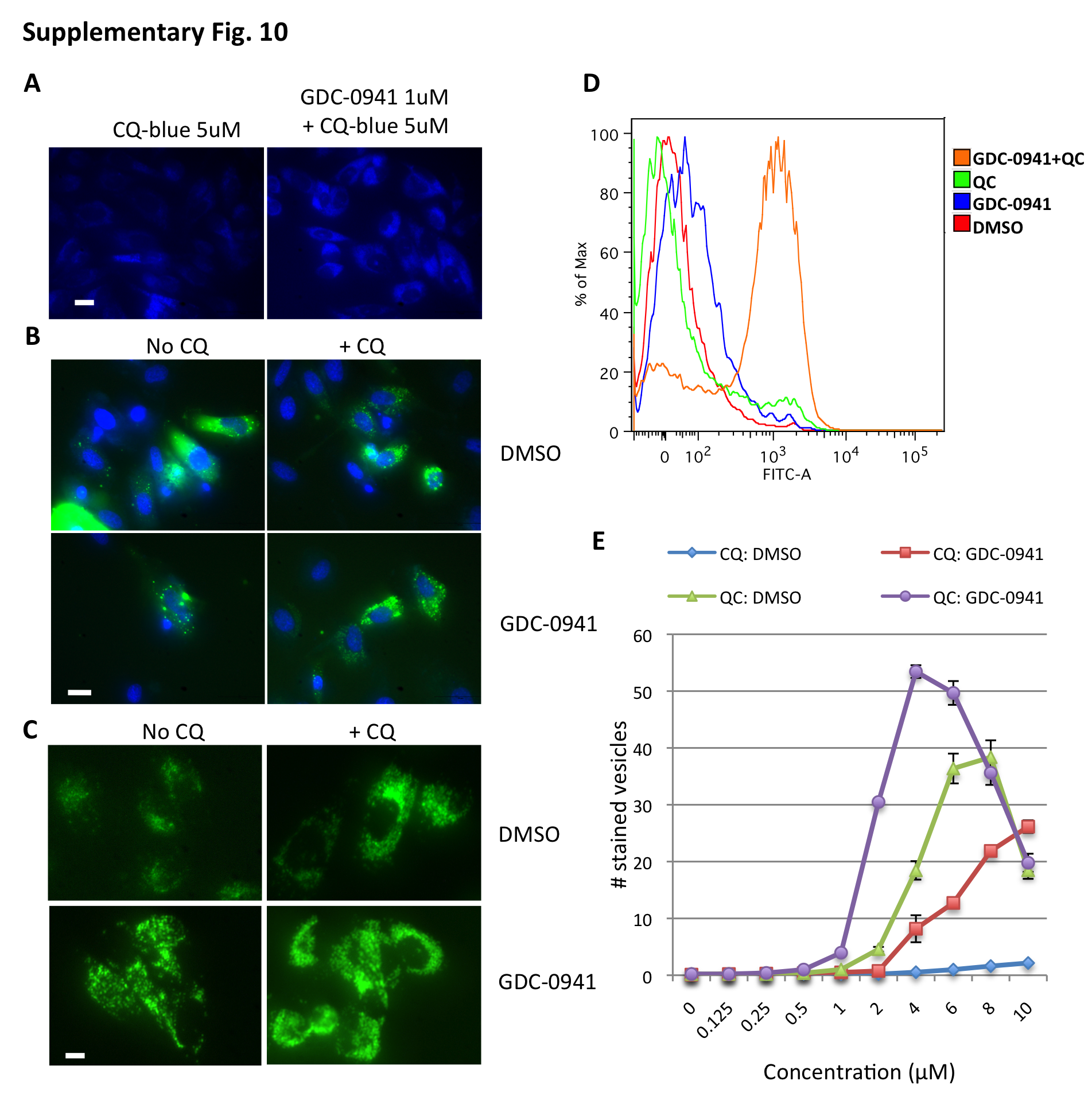

Supplement: Figure S10 — Fluorescent labeling of autophagic compartments. (A) LynxTagCQ-blue labels acidic vesicles in PC3 cells with a blue fluorescence. PC3 cells were treated with LynxTagCQ-blue (5 µM) +/− GDC-0941 (1 µM) for 24 hours and imaged with a 40× objective under microscope in the blue (DAPI) channel. (B) Labeling of AVs by eGFP-p62 in PC3 cells. PC3 cells were transfected with eGFP-p62 for 24 hours, then treated with GDC-0941 (1 µM) +/− CQ (10 µM) for 24 hours, stained with Hoechst 33342 and imaged with a 40× objective under microscope in green (FITC) and blue (DAPI) channels. Merged images in green and blue channels are shown. (C,D) Quinacrine-labeled AVs are fluorescent in the green channel and can be detected by OFACS. PC3 cells were treated with GDC-0941 (1 µM) +/− CQ (10 µM) for 24 hours, then stained with 1 µM quinacrine for 1 hour and imaged with a 40× objective under microscope in a green (FITC) channel (C). The same samples were then sonicated and analyzed by OFACS (D). Scale bars, 20 µm. (E) Dose response of CQ (AO+) and QC (FITC+) in inducing stained subcellular events by OFACS analysis. Error bars represent SEM (n = 3). (TIF) [file pone.0087707.s010.tif]

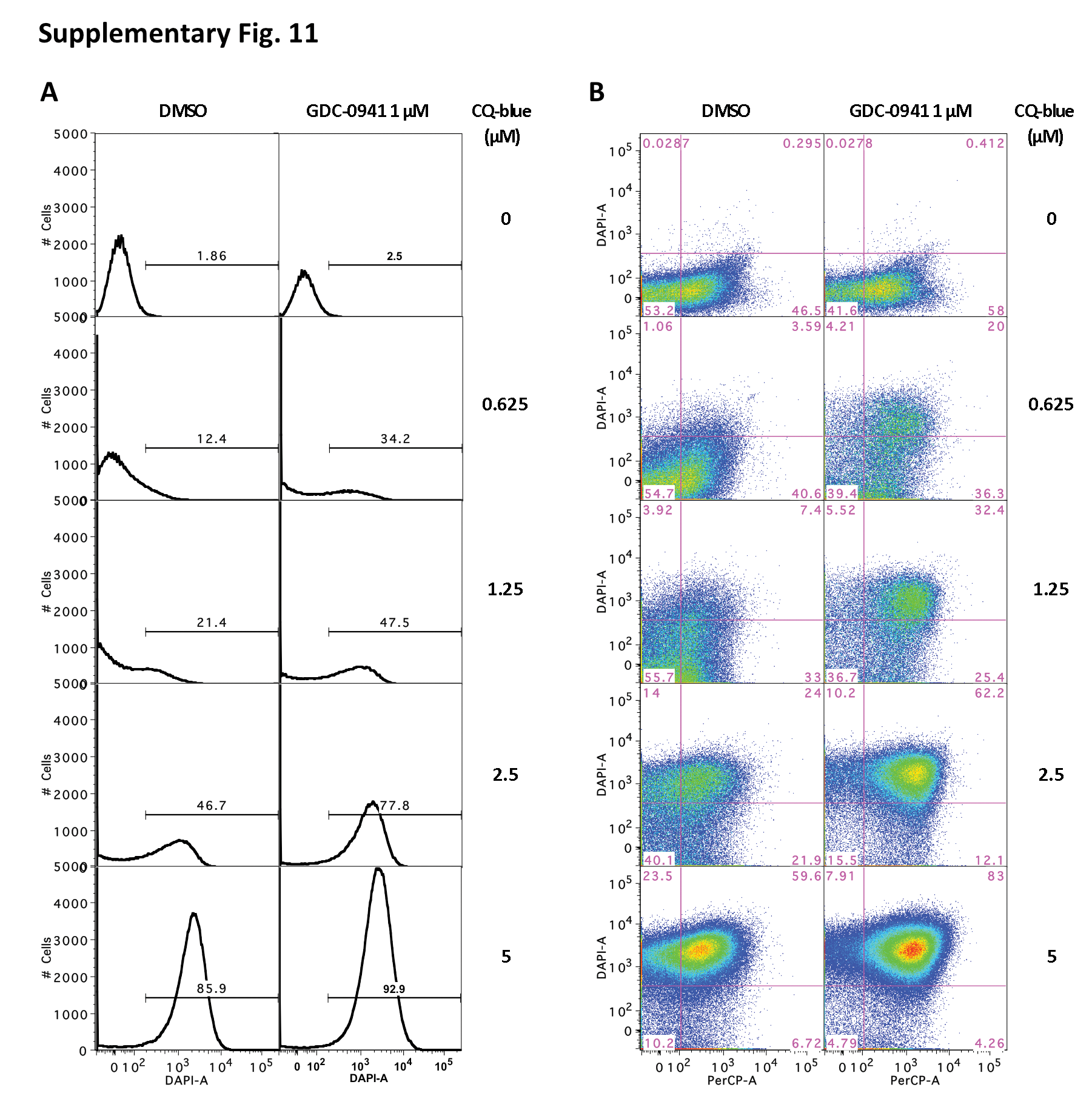

Supplement: Figure S11 — Co-localization of LynxTagCQ-blue labeled organelles with Acridine Orange. PC3 cells were treated +/− GDC-0941 (1 µM) with increasing concentrations of LynxTagCQ-blue (0–5 µM) for 24 hours, stained with AO, then sonicated and analyzed by OFACS. (A) Histograms in the blue (DAPI) channel. (B) Corresponding red (PerCP) vs. blue (DAPI) channel dot plots of the “organelle” population. (TIF) [file pone.0087707.s011.tif]

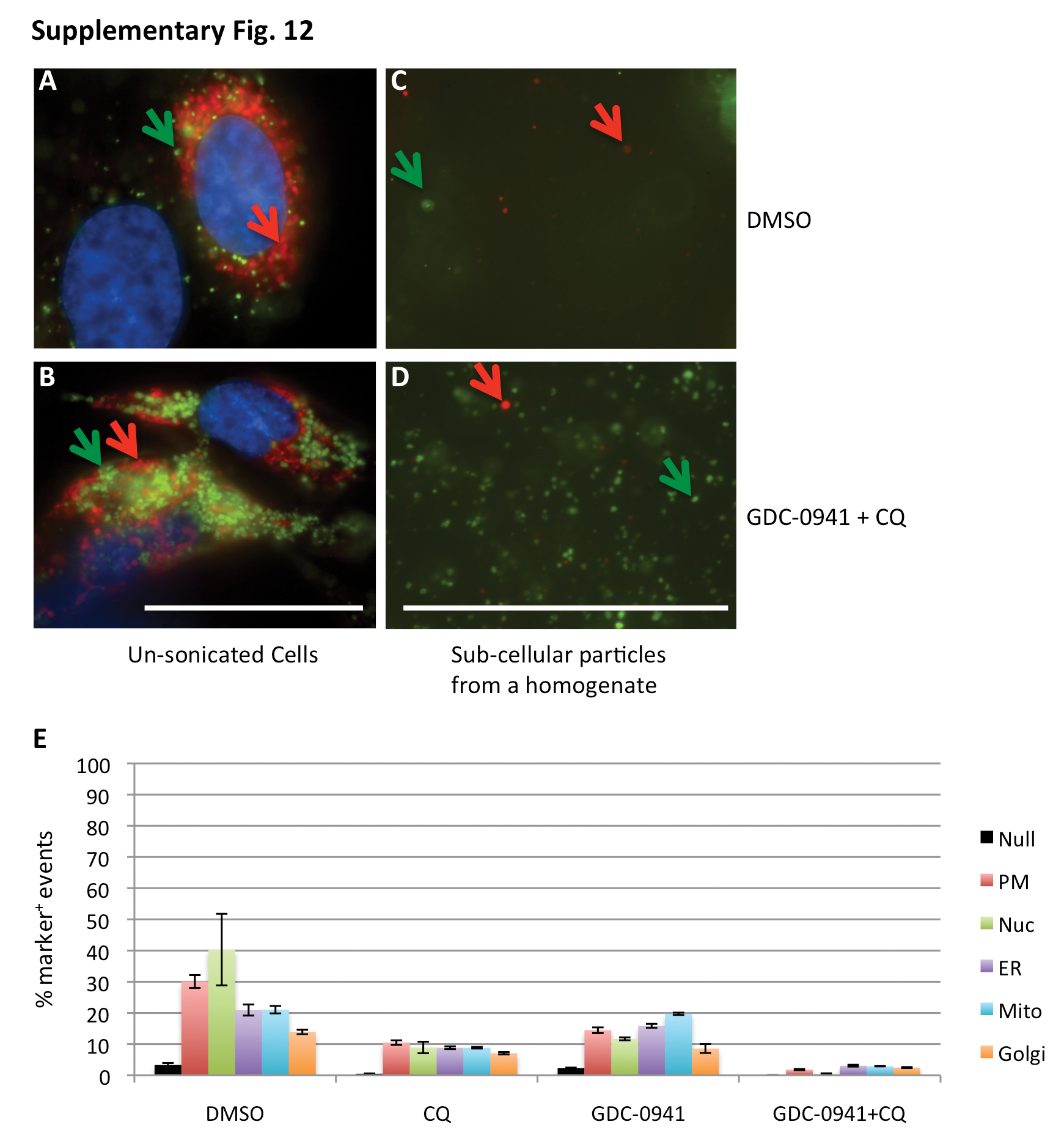

Supplement: Figure S12 — Contribution of subcellular membranes to the subcellular population in PC3 homogenates. (A–D) Representative images of mitochondria labeled with CellLight-Mitochondria-RFP kit (Invitrogen C10601) according to the manufacturer's protocol for 24 hours. Then cells were treated with 1 uM GDC-0941 +/− 10 uM CQ for 24 hours, then stained with 1 uM quinacrine for 45 minutes. Cells were imaged live with a 40× objective. Scale bars, 50 µm. RFP was detected in (550 nm ex/590 nm em) channel, quinacrine was detected in (488 nm ex/530 nm em) channel. After cell imaging, the same samples were analyzed by OFACS. Homogenates were centrifuged at 2000 g for 10 minutes in a glass bottom 96-well plate and imaged with a 100× objective using the same channel filters. RFP-labeled mitochondria (red arrow) and quinacrine-stained AVs (green arrow) are indicated in DMSO (Upper panel) and GDC-091+CQ (lower panel) treated samples. (E) Quantification of % marker positive events labeled with CellLight kits expressing the indicated organelle markers and QC. Numbers of RFP+QC−event were normalized to the number of RFP+ cells and calculated as the percentage of the normalized total subcellular events. Error bars represent SEM (n = 3). (TIF) [file pone.0087707.s012.tif]

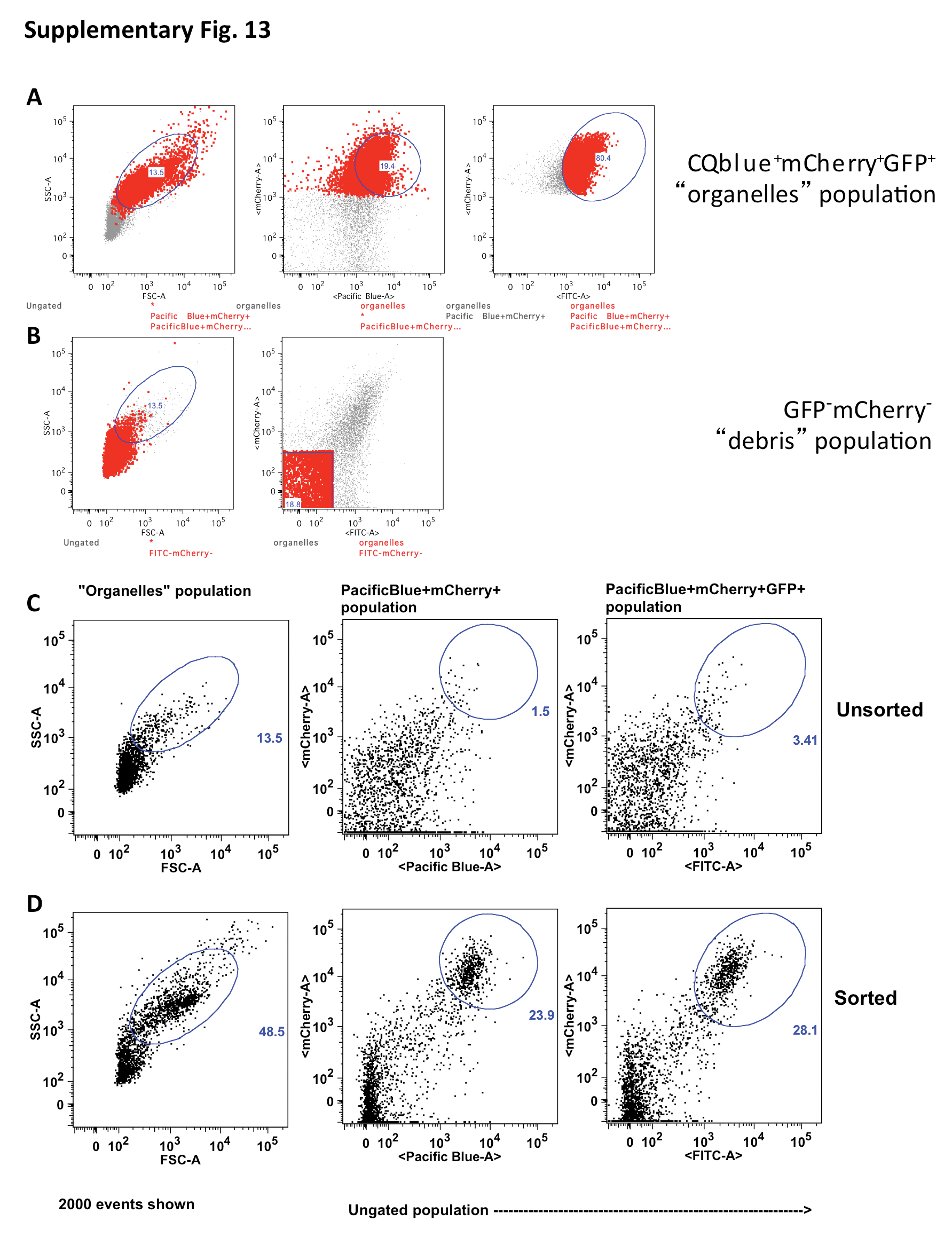

Supplement: Figure S13 — Flow cytometry sorting of AVs labeled with three different fluorophores. PC3 cells expressing mCherry-eGFP-LC3B were treated with 2 µM GDC-0941, 9 µM CQ and 1 µM LynxTag-CQ-blue (CQblue) for 24 hours, then sonicated according to the OFACS protocol. AVs labeled with three different fluorophores (mCherry, eGFP, LynxTag-CQblue) were subjected to flow cytometry sorting on a FacsAria flow cytometer sorter. CQ-blue was detected in PacificBlue channel (ex 405 nm/em 455 nm). Specific AVs (A) and debris (B) populations were established by backgating analysis and used as such for sorting. 2000 events from unsorted (C) and sorted (D) samples are shown. Populations for “organelles”, double fluorophore PacificBlue+mCherry+ or triple fluorophore PacificBlue+mCherry+GFP+ populations are circled with corresponding percentage in each population. (TIF) [file pone.0087707.s013.tif]
