# Supplementary material for: Novel Quantitative Autophagy Analysis by Organelle Flow Cytometry after Cell Sonication
Source: PLoS One. 2014 Jan 29;9(1):e87707. doi: 10.1371/journal.pone.0087707 (PMC3906200; doi:10.1371/journal.pone.0087707)
Supplement: Materials and Methods S1 — Supplementary Materials and Methods. (DOCX) [file pone.0087707.s014.docx]

**Novel quantitative autophagy analysis by organelle flow cytometry after cell sonication**

**Michael Degtyarev^1^, Mike Reichelt^2^ and Kui Lin^1^**

^1^ Department of Translational Oncology and ^2^ Department of Pathology, Genentech, South San Francisco, CA 94080 USA.

Correspondence should be addressed to Kui Lin at klin@gene.com.

**ImageStream assay**

PC3 cells expressing mCherry-eGFP-LC3B were treated with GDC-0941 (2 μM) +/- 10 μM CQ for 24 hours. Cells were dislodged with Trypsin/EDTA and resuspended in FACS buffer. In-sample cell number normalization was done by splitting cell suspension into two equal parts, then sonicating one part and combining it with the unsonicated half. AVs were analyzed by OFACS from aliquots of the same sample and compared to image flow cytometry analysis on ImageStream (Amnis Corporation).

**Organelle sorting**

PC3 cells expressing mCherry-eGFP-LC3B were treated with

2 μM GDC-0941, 9 μM CQ and 1 μM LynxTag-CQ-blue (CQblue) for 24 hours, then sonicated according to the OFACS protocol. AVs labeled with three different fluorophores (mCherry, eGFP, LynxTag-CQblue) were subjected to flow cytometry sorting on FacsAria flow cytometer sorter. CQ-blue was detected in the PacificBlue channel (ex 405nm/ex 455 nm). Specific AVs and debris populations were established by backgating analysis and used as such for sorting.
