# Supplementary material for: Novel Quantitative Autophagy Analysis by Organelle Flow Cytometry after Cell Sonication
Source: PLoS One. 2014 Jan 29;9(1):e87707. doi: 10.1371/journal.pone.0087707 (PMC3906200; doi:10.1371/journal.pone.0087707)
Supplement: Table S1 — Comparison of number of AVs per cell obtained by microscopic analysis and by OFACS analysis. (PDF) [file pone.0087707.s015.pdf]

Supplemental Table 1. Comparison of number of AVs per cell obtained by microscopic analysis and by OFACS analysis.

Cell line	Time (hours)	Microscopy eGFP⁺ Dots (number per cell) Treatment# 1	2	3	4	5	6	Correlation Coefficient	OFACS eGFP⁺ AVs (number per cell) Treatment# 1	2	3	4	5	6
HME	**6**	6	8	10	16	7	9	0.31	4	6	2	7	1	10
	**9**	7	7	14	23	6	11	0.79	5	7	2	29	4	18
	**12**	4	6	10	18	5	9	0.77	2	8	4	40	4	37
	**24**	2	4	7	22	4	14	0.96	7	34	6	177	8	128
PC3	**4**	2	4	2	6			0.87	3	3	2	4		
	**8**	2	8	7	15			0.75	4	3	2	7		
	**24**	2	12	6	33			0.98	1	4	1	26		
	**48**	2	15	4	53			1.00	2	13	2	55		

Treatment#	Drugs
**1**	DMSO
**2**	CQ 10 µM
**3**	GDC-0941 3 µM
**4**	GDC-0941 + CQ
**5**	GDC-0068 5 µM
**6**	GDC-0068 + CQ

HME (human mammary epithelial) cells stably expressing mCherry-eGFP-LC3B or PC3 cells stably expressing eGFP-LC3B were treated with GDC-0941 or GDC-0068 +/- CQ for indicated periods of time in 96-well plates. At stop time media was aspirated and cells fixed with 200 µl of 3.7% formaldehyde in Hank's Balanced Salt Solution (HBSS) for 15 min at room temperature, the solution was then replaced with 200 µl HBSS with 0.1 µg/ml Hoechst 33342 and the plates were stored at 4°C until analysis. Cells were imaged with a GE InCell2000 microscope (General Electric) with a 20x objective (HME) or a Nikon Eclipse TE300 microscope (Nikon) with a 40x objective (PC3). eGFP^{+} dot number per cell was analyzed by counting dots in 200 cells. Parallel un-fixed samples were analyzed by OFACS. Pearson correlation coefficients for sets of data at each time point obtained from each treatment by the two methods were calculated. Representative data from one of three independent experiments are shown.
